# To infinity and beyond: recent progress, bottlenecks, and potential of clonal seeds by apomixis

**DOI:** 10.1111/tpj.70054

**Published:** 2025-02-21

**Authors:** Bas Heidemann, Elias Primetis, Iris E. Zahn, Charles J. Underwood

**Affiliations:** ^1^ Department of Plant & Animal Biology, Radboud Institute for Biological and Environmental Sciences Radboud University Nijmegen the Netherlands; ^2^ Department of Chromosome Biology Max Planck Institute for Plant Breeding Research Carl‐von‐Linné‐Weg 10 50829 Cologne Germany

**Keywords:** clonal seeds, apomixis, meiosis, parthenogenesis, endosperm, hybrid vigor, plant breeding

## Abstract

Apomixis – clonal seed production in plants – is a rare yet phylogenetically widespread trait that has recurrently evolved in plants to fix hybrid genotypes over generations. Apomixis is absent from major crop species and has been seen as a holy grail of plant breeding due to its potential to propagate hybrid vigor in perpetuity. Here we exhaustively review recent progress, bottlenecks, and potential in the individual components of gametophytic apomixis (avoidance of meiosis, skipping fertilization by parthenogenesis, autonomous endosperm development), and sporophytic apomixis. The *Mitosis instead of Meiosis* system has now been successfully set up in three species (*Arabidopsis*, rice, and tomato), yet significant hurdles remain for universal bioengineering of clonal gametes. Parthenogenesis has been engineered in even more species, yet incomplete penetrance still remains an issue; we discuss the choice of parthenogenesis genes (*BABY BOOM*, *PARTHENOGENESIS*, *WUSCHEL*) and also how to drive egg cell‐specific expression. The identification of pathways to engineer autonomous endosperm development would allow fully autonomous seed production, yet here significant challenges remain. The recent achievements in the engineering of synthetic apomixis in rice at high penetrance show great potential and the remaining obstacles toward implementation in this crop are addressed. Overall, the recent practical examples of synthetic apomixis suggest the field is flourishing and implementation in agricultural systems could soon take place.

## Significant Statement

Most plant species reproduce by sex but a small, phylogenetically diverse group of species can clone themselves through seeds by apomixis. The engineering of apomixis in hybrid crops has revolutionary potential to fix hybrid vigor. Here we review recent progress, bottlenecks, and potential in the individual components of apomixis which include the avoidance of meiosis, skipping fertilization by parthenogenesis and autonomous endosperm development.

## INTRODUCTION

Apomixis – asexual seed production – is an alternative strategy to sexual reproduction that is employed in about 400 plant species (Ozias‐Akins & Van Dijk, 2007; Wang & Underwood, 2023). Although relatively rare (less than 0.1% of plant species are apomictic), it is present in phylogenetically diverse plant species, indicating the likely recurrent evolution of apomixis (Hojsgaard et al., 2014). Based on cytological observations of apomixis in divergent species it has been concluded that several different types of apomixis exist, further substantiating apomixis is a recurrently evolving trait (Koltunow & Grossniklaus, 2003). The introduction of apomixis into sexual crops has revolutionary potential in agriculture to fix hybrid genotypes through seeds and thereby fundamentally change the supply and availability of hybrid seeds worldwide (Underwood & Mercier, 2022).

**Figure 1:**
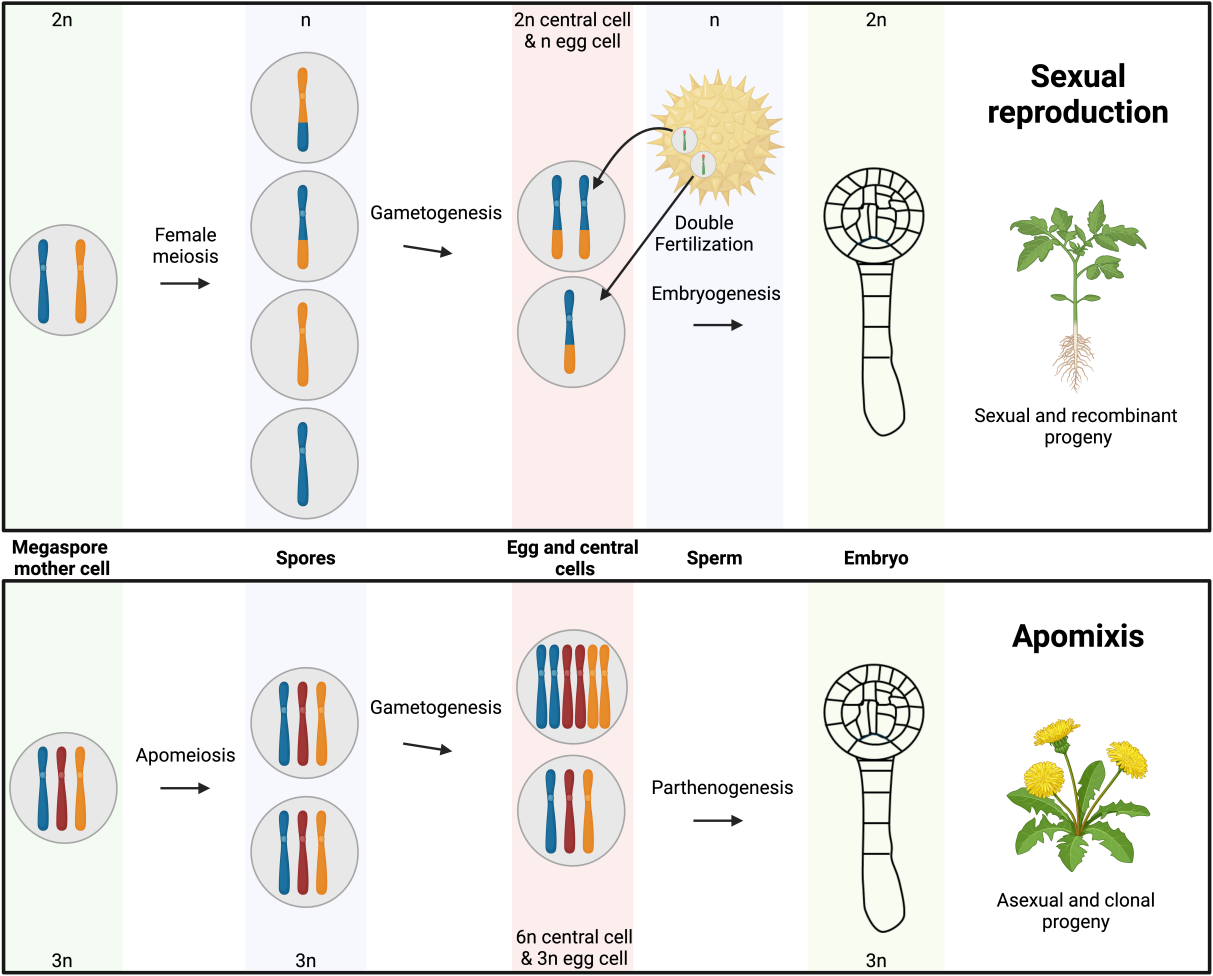
Schematic of sexual and apomictic reproduction at the cellular level. Sexual reproduction in tomato (*Solanum lycopersicum*) is depicted in the top half of the figure and apomictic reproduction in dandelion (*Taraxacum officinale*) is depicted in the bottom half of the figure. In sexual reproduction in tomato, the chromosomes replicate in the megaspore mother cell and subsequently meiosis takes place. This leads to the production of four recombinant, haploid cells (the megaspores), three of which degenerate, while one survives and enters gametogenesis (three rounds of mitotic division). The mature female gametophyte encompasses one haploid egg cell, one diploid central cell, three haploid antipodals (not shown) and two haploid synergids (not shown). The process of double fertilization of the egg cell and central cell leads to a diploid embryo and a triploid endosperm (not shown). In apomictic reproduction in dandelion, the chromosomes replicate in the megaspore mother cell and subsequently a diplospory apomeiosis division takes place. This leads to the production of two non‐recombinant, triploid cells (the megaspores). One megaspore degenerates and one enters gametogenesis. The mature female gametophyte encompasses one triploid egg cell, one hexaploid central cell, three triploid antipodals (not shown) and two triploid synergids (not shown). The egg cell enters embryogenesis by parthenogenesis (triggered by the *ToPAR* gene) giving rise to a triploid embryo while the central cell autonomously begins endosperm development giving rise to a hexaploid endosperm (not shown). Created in Biorender. Underwood, C. (2025) https://Biorender.com/a96z426

To comprehend the intricacies of apomixis it is insightful to consider the two defining features of flowering plant sexual reproduction: meiosis and double fertilization (Dresselhaus et al., 2016; Mercier et al., 2015) (Figure 1). Meiosis, a conserved cell division in the reproduction of eukaryotes, takes place in plants in the male and female floral organs. In cells with a meiotic destiny, a pre‐meiotic S phase leads to the replication of chromosomes. Distinct to mitosis, during the first meiotic prophase programmed DNA double‐strand breaks are made, homologous chromosomes pair, and homologous recombination takes place. Subsequently, homologous chromosomes segregate and only in the second meiotic division do the sister chromatids segregate. As a result, haploid, recombinant cells are produced that may enter gametogenesis to generate two sperms (encased in the pollen) on the male side, and a central cell and an egg cell (encased in the female gametophyte) on the female side. During double fertilization, one sperm fertilizes the egg, giving rise to the embryo, while the fertilization of the central cell gives rise to the endosperm (a nourishing tissue for the embryo). The developing embryo and endosperm are surrounded by a maternal‐derived seed coat.

Ultimately, for clonal seed production by apomixis to evolve, the processes of meiosis and egg cell fertilization must be avoided (Underwood & Mercier, 2022). One route to this is sporophytic apomixis (also referred to as adventive embryony and nucellar embryony) which is the formation of a somatic embryo from a non‐gametic lineage that co‐occupies the seed with a sexual embryo (Lakshmanan & Ambegaokar, 1984). Sporophytic apomixis occurs naturally in *Citrus* and *Mangifera* (commonly referred to as mango) and leads to multiple embryos within a single seed – so‐called polyembryony (Lakshmanan & Ambegaokar, 1984). Alternatively, gametophytic apomixis requires the modification and/or skipping of the sexual processes (female meiosis and double fertilization) themselves (Nogler, 1984).

Among many other genera, gametophytic apomixis occurs naturally in the wild grass genera *Cenchrus*/*Pennisetum* and the Asteraceae family genera *Taraxacum, Hieracium*, and *Pilosella* (Ozias‐Akins & Van Dijk, 2007). Broadly speaking there are two natural routes to skip female meiosis, namely, apospory and diplospory (Koltunow & Grossniklaus, 2003). Apospory is the development of the female gametophyte from the sporophyte (i.e., somatic cells of the ovule) without the formation of spores and occurs naturally in *Pennisetum* and *Pilosella* (Bicknell et al., 2023; Huo et al., 2009). On the other hand, a diplospory division leads to the production of two clonal spores, one of which enters gametogenesis, and occurs naturally in *Taraxacum* (by meiotic diplospory) (Figure 1) and *Hieracium* (by mitotic diplospory) (Bicknell et al., 2023; Cornaro et al., 2024; Dijk & Bakx‐Schotman, 2004). In all cases of gametophytic apomixis, a cell with egg cell fate inside the female gametophyte must then be triggered to enter embryogenesis by a process known as parthenogenesis (Underwood & Mercier, 2022). The genetic basis of parthenogenesis has been identified in *Pennisetum* and *Taraxacum* (Conner et al., 2015; Underwood et al., 2022). To support embryo development, most apomictic species initiate the formation of the endosperm by fertilization of the central cell by a sperm (known as pseudogamy) (Nogler, 1984). However, in some species, including dandelion (*Taraxacum officinale*), the requirement of central cell fertilization to trigger endosperm development has been lost. Through this autonomous endosperm development, these species can reproduce completely independent of fertilization (Hands et al., 2016).

Engineering of apomixis has long been considered as a holy grail for the propagation of hybrid genotypes in crop plants (Jefferson, 1994; Spillane et al., 2004; Underwood & Mercier, 2022). Besides the engineering of complete synthetic apomixis systems in crops, individual components of apomixis could have applications in crop breeding. For example, the skipping of meiosis alone can be used to generate non‐recombinant double‐cross hybrid seed which can be used for polyploid genome design (Wang et al., 2024). In addition, the skipping of fertilization by parthenogenesis can be used as a double haploid technology to rapidly fix recombinant haplotypes (Jacquier et al., 2020; Quiroz et al., 2024), while autonomous endosperm could be used as a strategy to ensure grain filling in harsh environmental conditions (Hands et al., 2016).

Natural and synthetic apomixis have been extensively reviewed (Goeckeritz et al., 2024; Khanday & Sundaresan, 2021; Mahlandt et al., 2023; Qu et al., 2024; Underwood & Mercier, 2022; Vijverberg et al., 2019; Xiong et al., 2023; Yin et al., 2022), so here we specifically focus on significant advances related to apomixis in the last 3 years (from January 2022 until January 2025) and bottlenecks, possible solutions and potential applications for the future (see Box 1 for a summary of the key review contents).

Box 1 **Review summary**

- Apomixis is a complex multigenic trait that cannot be introduced into sexual crops with a single straightforward mutation.
- Discovery and functional analysis of more natural apomixis genes will aid future engineering of apomeiosis, parthenogenesis, and autonomous endosperm in crops.
- *Mitosis instead of Meiosis* (*MiMe*) leads to clonal male and female gametes in *Arabidopsis*, rice, and tomato, yet application in other crops is not straightforward due to complex genetics of skipping the second meiotic division.
- Three independent insertions in *PARTHENOGENESIS* gene promoters and three independent insertions in *RWP* gene promoters appear to cause the evolution of apomixis in natural apomicts.
- Although high clonal seed rates and almost normal fertility have been achieved in rice, combining these two traits in a dicot plant is still challenging.

## RECENT ADVANCES IN SYNTHETIC APOMIXIS (JANUARY 2022–JANUARY 2025)

**Figure 2:**
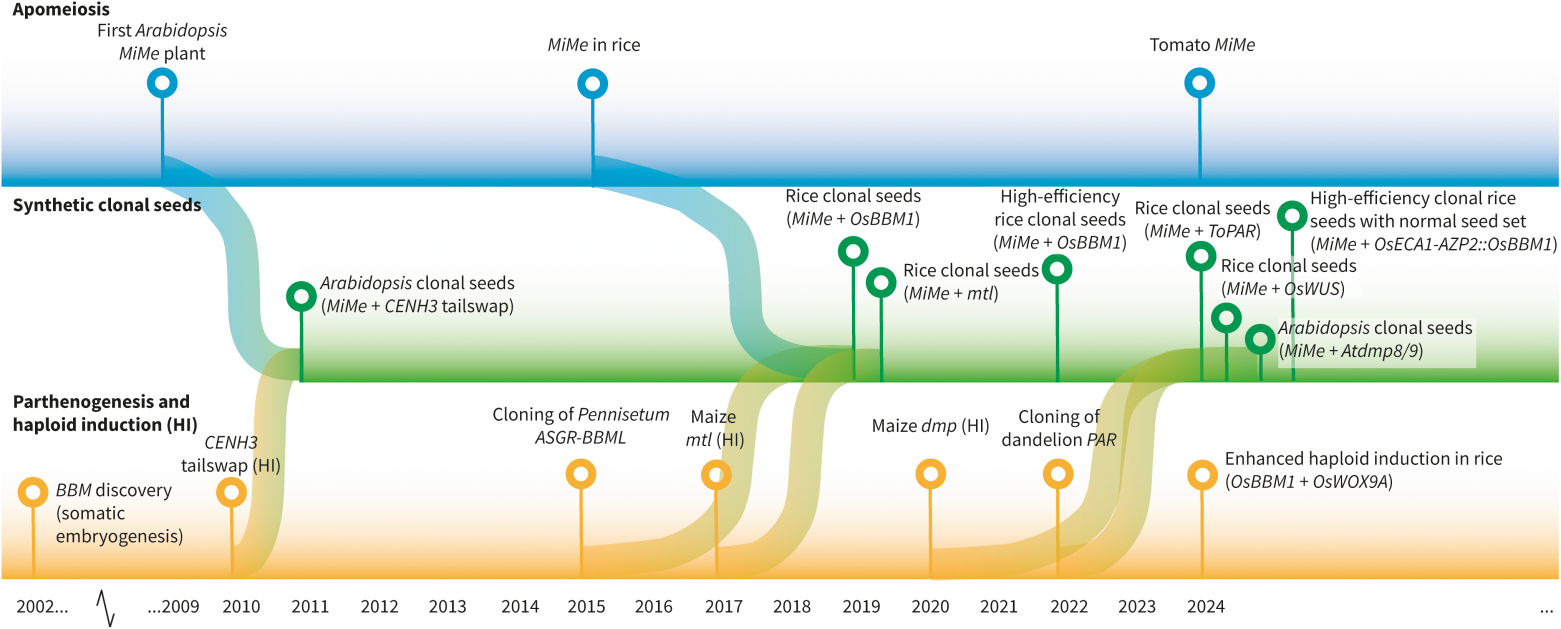
Milestones in synthetic apomixis research. Timeline of milestones in apomeiosis research (blue), parthenogenesis and haploid induction (HI) (yellow), which are combined in studies that bioengineer clonal seeds (green). The different height of the green markers is for esthetic purposes and has no meaning.

The recent advances in synthetic apomixis are classified into four categories: (1) skipping of meiosis, skipping fertilization by (2) parthenogenesis or (3) sporophytic apomixis, and (4) autonomous endosperm development. In recent years, several studies in rice (*Oryza* sativa) have combined multiple elements of apomixis to engineer synthetic apomixis systems that facilitate clonal seed production, which will be further elaborated upon independently in a fifth section. An overview of major developments in apomixis research is shown in Figure 2.

### Skipping meiosis

The *MiMe* system, standing for *Mitosis instead of Meiosis*, was first developed in *Arabidopsis* in 2009 using a combination of mutations in *SPORULATION 11–1* (*AtSPO11‐1*), *RECOMBINATION 8* (*AtREC8*) and *OMISSION OF SECOND DIVISION 1* (*AtOSD1*). The *Atspo11‐1 Atrec8 Atosd1* triple mutant generates clonal male and female gametes at high penetrance (D'Erfurth et al., 2009). *MiMe* relies upon the consecutive elimination of meiotic recombination initiation (*Atspo11‐1*), sister chromatid cohesion/monopolar orientation of kinetochores (*Atrec8*), and the second meiotic division (*Atosd1*). Since the work in *Arabidopsis*, a *MiMe* system has been developed in rice with the mutant combination *Ospair1 Osrec8 Ososd1* which modifies the same three aspects of meiosis (Mieulet et al., 2016). The combination of *MiMe* in rice with maternal haploid induction (Liu et al., 2023; Wang et al., 2019) or parthenogenesis (Dan et al., 2024; Huang, Meng, et al., 2024; Khanday et al., 2019; Liu et al., 2023; Song, Wang, Ji, et al., 2024; Vernet et al., 2022; Wei et al., 2023), has resulted in the first crop synthetic apomixis systems.

A recent study led to the development of a *MiMe* system in tomato (*Solanum lycopersicum*); the first *MiMe* system in a vegetable and a dicot crop. In this *MiMe* system, mutations of the *SlSPO11‐1*, *SlREC8*, and *TARDY ASYNCHRONOUS MEIOSIS* (*SlTAM*) genes were combined, resulting in male and female unreduced clonal gametes (Wang et al., 2024). The system was first established in the Micro‐Tom background and later applied in three hybrid tomato genotypes; a Moneyberg‐TMV × Micro‐Tom (MbTMV‐MT) model hybrid, a ‘Funtelle’ date‐tomato commercial hybrid, and a ‘Maxeza’ truss tomato commercial hybrid (Wang et al., 2024). Selfing of the diploid *MiMe* plants leads to tetraploid offspring at high penetrance (93%) and the absence of meiotic recombination which was demonstrated by whole genome sequencing (Wang et al., 2024). However, in some cases, likely due to SPO11‐1‐independent DNA double‐strand breaks, chromosome truncations were observed in offspring plants indicating the tomato *MiMe* system could be further improved (Wang et al., 2024). Additionally, by hybridizing *MiMe* plants generated in two different hybrid backgrounds, a new breeding paradigm called polypoid genome design was developed. Through the fusion of a clonal sperm from one hybrid parent with the clonal egg of another hybrid parent, '4‐haplotype' plants (non‐recombinant double‐cross hybrid plants) were developed that contained the complete genetic information of both parents – the first report in a plant or animal (Wang et al., 2024). In summary, this is the first study in which the *MiMe* system was successfully applied in a dicot crop and demonstrated that hybrid organisms that produce clonal gametes can be harnessed to perform precise polyploid genome engineering (Awan et al., 2024; Wang et al., 2024).

### Parthenogenesis

Skipping egg cell fertilization by parthenogenesis initiates the development of an embryo directly from an unfertilized egg cell, thereby bypassing the need for a male gamete for embryogenesis. To date, *Pennisetum squamulatum APOSPORYSPECIFIC GENOMIC REGION‐BABYBOOM‐LIKE* (*PsASGR‐BBML*) and dandelion *PARTHENOGENESIS* (*ToPAR*) are the only identified natural parthenogenesis genes (Conner et al., 2015; Underwood et al., 2022). BABY BOOM (BBM), an APETALA 2 (AP2)/ETHYLENE RESPONSIVE FACTOR (ERF) domain transcription factor, had been known as an embryogenic factor for many years before its role in parthenogenesis (Boutilier et al., 2002). However, no role for ToPAR, a K2‐2 zinc finger domain protein with an EAR motif, in embryogenesis was previously known (Underwood et al., 2022). Two particularly sensational developments in recent years in rice include the combination of *MiMe* with egg cell expression of *OsBBM1* or *ToPAR* for synthetic apomixis and clonal seed production (Khanday et al., 2019; Song, Wang, Ji, et al., 2024). In addition, *WUSCHEL* (*WUS*) has recently been demonstrated to induce parthenogenesis in rice (Huang, Meng, et al., 2024). In contrast to *BBM* and *PAR*, *WUS* is not a factor cloned from a natural parthenogenesis system, yet is known to be involved in several aspects of plant development, including the induction of somatic embryogenesis and embryo development (Jha & Kumar, 2018; Zuo et al., 2002). An exhaustive overview of parthenogenesis factors investigated in a variety of species is presented in Table 1.

**Table 1:** Promoters and genes to induce parthenogenesis

Species	Female accession/cultivar	Female mutation status^a^	Female parent construct^b^		Male parent^e^	Phenotype^f^	Reference
			Promoter^c^	Gene^d^			
*Arabidopsis thaliana*	Col‐0	WT	AtEC1.2	*PsASGR‐BBMLgeno*	*	No haploid seed production detected	Conner et al. (2017)
	Col‐0	WT	AtEC1.2	*PsASGR‐BBML*	*	No haploid seed production detected	Conner et al. (2017)
	Col‐0	WT	AtEC1.2en‐AtEC1.1:AMV	*BnBBM1*	*	1.5% parthenogenic embryo formation; 0.4% haploid seed production	Chen et al. (2022)
	Col‐0	WT	AtEC1.2en‐AtEC1.1:AMV	*BnBBM1‐GR*	*	5.4% parthenogenic embryo formation	Chen et al. (2022)
	Col‐0	WT	AtRPS5A:AMV	*BnBBM1*	*	6.2% parthenogenic embryo formation; 0% haploid seed production	Chen et al. (2022)
	Col‐0	WT	AtEC1.2	*AtBBMgeno‐GFP*	*	No transgene expression in the egg cell, no parthenogenesis	Liu, Han, et al. (2024)
	Col‐0	WT	AtEC1.2	*AtBBM‐GFP*	*	Weak transgene expression in egg cell, no parthenogenesis	Liu, Han, et al. (2024)
	Col‐0	WT	AtEC1.2	*OsBBM1geno‐RFP*	*	Expression of transgene in egg cell, but no parthenogenesis	Liu, Han, et al. (2024)
	Col‐0	WT	AtEC1.2	*OsBBM1‐RFP*	*	Expression of transgene in egg cell, but no parthenogenesis	Liu, Han, et al. (2024)
	Col‐0	WT	AtEC1.2	*AtBBM* codon optimized	*	1.98% parthenogenic embryo formation until two‐cell stage	Liu, Han, et al. (2024)
	Col‐0	WT	AtEC1.2	*AtBBM‐OsBBM1* chimera	*	Two‐cell parthenogenic embryos can be observed, not quantified	Liu, Han, et al. (2024)
	Col‐0	*Atms1*	AtEC1.2en‐AtEC1.1:AMV	*BnBBM1*	Col‐0	0.1%–1.0% haploid progeny	Chen et al. (2022)
	Col‐0	*Atms1*	AtEC1.2en‐AtEC1.1:AMV	*BnBBM1*	*FAST‐Red* in Col‐0	0.4%–2.8% haploid progeny	Chen et al. (2022)
	Col‐0	*Atms1*	AtEC1.2en‐AtEC1.1:AMV	*BnBBM1*	*Atdmp8/9; FAST‐Red*	3.7% haploid progeny	Chen et al. (2022)
	Col‐0	WT	AtEC1.2en‐AtEC1.1:AMV	*BnBBM1*	*Atdmp8/9; FAST‐Red*	5.9%–8.6% haploid progeny	Chen et al. (2022)
	Col‐0	WT	AtEC1.2	*AtBBM*	*Atdmp8/9*	1.80% haploid progeny	Liu, Han, et al. (2024)
	Col‐0	*Atrkd5‐3*	AtEC1.2	*AtBBM*	*Atdmp8/9*	2.44% haploid progeny	Liu, Han, et al. (2024)
	Col‐0	*Atdad1*	AtEC1.2	*AtBBM* codon optimized	*Atdmp8/9*	2.17% haploid progeny	Liu, Han, et al. (2024)
	Col‐0	*Atdad1*	AtEC1.2	*AtBBM‐OsBBM1* chimera	*Atdmp8/9*	5.09% haploid progeny	Liu, Han, et al. (2024)
	Col‐0	*Atrkd5‐3*	AtEC1.2	*AtBBM*	*Atrkd5‐3*	1.60% parthenogenic zygote formation; 0% mature parthenogenic seeds	Liu, Han, et al. (2024)
*Brassica napus*	DH12075	WT	AtEC1.2en‐EC1.1:AMV	*BnBBM1*	*RPS5A:AMV:GFP*	0.1% haploid progeny	Chen et al. (2022)
	DH12075	WT	AtEC1.2en‐EC1.1:AMV	*BnBBM1*	*Bndmp; FAST‐Red*	1.8%–2.0% haploid progeny	Chen et al. (2022)
*Pennisetum glaucum*	IA4X	WT	PsASGR‐BBML	*PsASGR‐BBMLgeno*	*	35%–36% haploid embryo formation; decreased germination rate and seed set	Conner et al. (2015)
*Nicotiana tabacum*	Xanthi NN	WT	AtEC1.2	*PsASGR‐BBML*	*	<1% haploid progeny	Zhang et al. (2020)
	Xanthi NN	WT	AtRKD2	*PsASGR‐BBML*	*	9.3% haploid progeny in T1 and 2.7%–27.3% haploid progeny in T2	Zhang et al. (2020)
*Solanum lycopersicum*	Microtom	WT	AtEC1.2en‐EC1.1:AMV	*BnBBM1*	*	1.4% haploid progeny	Chen et al. (2022)
	Microtom	WT	AtEC1.2en‐EC1.1:AMV	*BnBBM1*	*Sldmp*	1.5%–5.9% haploid progeny	Chen et al. (2022)
	Microtom	WT	AtEC1.2en‐EC1.1:AMV	*BnBBM1*	*Sldmp; FAST‐Red*	18% haploid progeny	Chen et al. (2022)
*Taraxacum officinale*	A68	*LoP mutant*	ToPAR	*Lssex*	*	4/8 lines complement *loss‐of‐parthenogenesis* (*LoP*) mutant phenotype	Underwood et al. (2022)
	A68	*LoP mutant*	AtEC1.1	*ToPAR*	*	5/9 lines complement *LoP* mutant phenotype	Underwood et al. (2022)
*Lactuca sativa*	Legacy (iceberg type)	WT	AtEC1.1	*ToPAR*	*	7/7 lines produce parthenogenic embryo‐like structures	Underwood et al. (2022)
*Setaria italica*	*Ci846*	WT	AtEC1.2	*ToPAR*	*	1.4%–10.2% haploid progeny	Huang, Liang, et al. (2024)
*Oryza sativa*	Nipponbare	WT	PsASGR‐BBML	*PsASGR‐BBMLgeno*	*	87% of transgenic lines produce haploid embryos	Conner et al. (2017)
	Nipponbare	WT	PsASGR‐BBML	*PsASGR‐BBML*	*	25% of transgenic lines produce haploid embryos	Conner et al. (2017)
	Nipponbare	WT	AtEC1.2	*PsASGR‐BBMLgeno*	*	89% of transgenic lines produce haploid embryos	Conner et al. (2017)
	Kitaake	WT	AtEC1.2	*OsBBM1*	*	12% of ovules produce parthenogenic embryonic structures; 5.8%–10.5% haploid progeny in T1	Khanday et al. (2019)
	CY84	WT	AtEC1.2	*OsBBM2*	*	No parthenogenesis observed	Wei et al. (2023)
	CY84	WT	AtEC1.2	*OsBBM3*	*	No parthenogenesis observed	Wei et al. (2023)
	CY84	WT	AtEC1.2	*OsBBM4*	*	3.2% haploid progeny with a seed set of 21.1%–82.6%	Wei et al. (2023)
	Kitaake	WT	OsECA1	*OsBBM1*	*	0% parthenogenic haploid progeny	Vernet et al. (2022)
	Kitaake	WT	AtEC1.2	*OsBBM1*	*	10%–29% haploid progeny	Ren et al. (2024)
	Kitaake	WT	AtEC1.2	*OsWOX9A*	*	0% parthenogenic haploid progeny	Ren et al. (2024)
	Kitaake	WT	AtEC1.2; AtEC1.2	*OsBBM1*; *OsWOX9A*	*	3.70%–48.21% haploid progeny in hemizygous T1; 86%–91% haploid frequency in homozygous T2	Ren et al. (2024)
	BRS‐CIRAD 302	*Ospair 1 Osrec8 Ososd1*	OsECA1	*OsBBM1*	*	83%–100% diploid clonal progeny in T1, with a panicle fertility of 33%–35%	Vernet et al. (2022)
	Kitaake	*Ospair 1 Osrec8 Ososd1*	OsECA1	*OsBBM1*	*	55–84% clonal progeny in T1, with a panicle fertility of 59–82%	Vernet et al. (2022)
	XS134	*Ospair1 Osrec8 Ososd1 Ossd1*	OsECA1	*OsBBM1*	*	5.9%–97.1% clonal progeny in T1, with a seed set of 13.9%–51.0%	Song, Li, Chen, et al. (2024)
	YY4949	*Ospair1 Osrec8 Ososd1 Ossd1*	OsECA1	*OsBBM1*	*	5.7%–88.2% clonal progeny in T1, with a seed set of 5.7%–88.2%	Song, Li, Chen, et al. (2024)
	YY1538	*Ospair1 Osrec8 Ososd1 Ossd1*	OsECA1	*OsBBM1*	*	17.1%–85.4% clonal progeny in T1, with a seed set of 17.1%–85.4%	Song, Li, Chen, et al. (2024)
	XS134	*Ospair1 Osrec8 Ososd1 Ososd1*	OsECA1:AZP1‐10 x GCN4	*OsBBM1*	*	3.6%–95.1% clonal progeny in T1	Song, Li, Chen, et al. (2024)
	YY4949	*Ospair1 Osrec8 Ososd1 Ososd1*	OsECA1:AZP1‐10 x GCN4	*OsBBM1*	*	7.4%–91.2% clonal progeny in T1	Song, Li, Chen, et al. (2024)
	XS134	*Ospair1 Osrec8 Ososd1 Ososd1*	OsECA1:AZP2‐10 x GCN4	*OsBBM1*	*	4.7%–100% clonal progeny in T1, with a seed set of 18.6%–91.2%	Song, Li, Chen, et al. (2024)
	YY4949	*Ospair1 Osrec8 Ososd1 Ososd1*	OsECA1:AZP2‐10 x GCN4	*OsBBM1*	*	4.1%–100% clonal progeny in T1, with a seed set of 11.3%–83.4%	Song, Li, Chen, et al. (2024)
	YY1538	*Ospair1 Osrec8 Ososd1 Ososd1*	OsECA1:AZP2‐10 x GCN4	*OsBBM1*	*	73.1%–100% clonal progeny in T1, with a seed set of 12.4%–83.8%	Song, Li, Chen, et al. (2024)
	Kitaake	*Ospair 1 Osrec8 Ososd1*	AtEC1.2	*OsBBM1*	*	11.1%–29.2% clonal progeny in T1	Khanday et al. (2019)
	BRS‐CIRAD 302	*Ospair 1 Osrec8 Ososd1*	AtEC1.2	*OsBBM1*	*	80%–100% clonal progeny in T1, with a panicle fertility of 27%–33%	Vernet et al. (2022)
	Kitaake	*Ospair 1 Osrec8 Ososd1*	AtEC1.2	*OsBBM1*	*	55%–84% clonal progeny in T1, with a panicle fertility of 74%–81%	Vernet et al. (2022)
	YY4	*Ospair 1 Osrec8 Ososd1*	AtEC1.2	*OsBBM1*	*	90%–96% clonal progeny with a seed set of 44%–51%	Dan et al. (2024)
	XS134	*Ospair1 Osrec8 Ososd1 Ossd1*	AtEC1.2	*OsBBM1*	*	1.0%–96.2% clonal progeny in T1, with a seed set of 11.9%–90.9%	Song, Li, Chen, et al. (2024)
	YY4	*Ospair 1 Osrec8 Ososd1*	AtEC1.2; AtMYB98_AtDD1_OsECA1‐like1	*OsBBM1*; *AtWUS*	*	66%–97% clonal progeny with a seed set of 33%–68%	Dan et al. (2024)
	CY84	*Ospair 1 Osrec8 Ososd1*	AtEC1.2	*OsBBM4*	*	1.67% clonal progeny, with a seed set of 80%	Wei et al. (2023)
	CY84	*Ospair 1 Osrec8 Ososd1*	AtEC1.2	*OsWUSgeno*	*	0.5%–21.7% clonal progeny, with a seed set of 80%	Huang, Liang, et al. (2024)
	JHY7245	*Ospair 1 Osrec8 Ososd1*	AtEC1.1	*ToPAR*	*	42%–67% apomictic progeny in T1, with a seed set of 74%–82%	Song, Wang, Ji, et al. (2024)
	JFY2	*Ospair 1 Osrec8 Ososd1*	AtEC1.1	*ToPAR*	*	43%–62% apomictic progeny in T1, with a seed set of 67%–74%	Song, Wang, Ji, et al. (2024)
	JFY2	*Ospair 1 Osrec8 Ososd1 Osbc1*	AtEC1.1	*ToPAR*	*	56% apomictic progeny in T1, with a seed set 73%	Song, Wang, Ji, et al. (2024)
	JFY2	*Ospair1 Osrec8 Ososd1 Ossd1*	AtEC1.1	*ToPAR*	*	42% apomictic progeny in T1, with a seed set 78%	Song, Wang, Ji, et al. (2024)
*Zea mays*	Hi Type II hybrid	WT	PsASGR‐BBML	*PsASGR‐BBMLgeno*	*	47% of transgenic lines produce haploid embryos; strongly decreased pollen viability	Conner et al. (2017)
	Hi Type II hybrid	WT	AtEC1.2	*PsASGR‐BBMLgeno*	*	80% of transgenic lines produce haploid embryos; strongly decreased pollen viability	Conner et al. (2017)
	ZC01	WT	CRISPRa using VP64 and p65‐HSF directed to ZmBBM2	*ZmBBM2*	*	0.4%–3.55% haploid seeds	Qi et al. (2023)
	B104	WT	AtEC1.2	*ZmBBM1*	*	65% haploid progeny in T1	Skinner et al. (2023)
	X08D492	WT	ZmEC	*ZmBBM2*	PH1V69‐CFP	9.3% parthenogenic haploid induction	Ye et al. (2024)
	X08D492	WT	PvEC	*ZmBBM2*	PH1V69‐CFP	17.3% parthenogenic haploid induction	Ye et al. (2024)
	PHR03/PH184C	WT	PvEC	*ZmBBM2*	PH1V69‐CFP	18.4%–18.9% parthenogenic haploid induction	Ye et al. (2024)
	PHR03/PH184C	WT	PvEC;PvEC	*ZmBBM2*; *ZmCYCD2*	PH1V69‐CFP	24.6% parthenogenic haploid induction	Ye et al. (2024)

*Note*: Combinations of promoters and genes investigated to induce parthenogenesis, with their respective phenotypes.

^a^ The column “Female mutation status” describes any genetic mutations present in the background of female parent; for example, the triple mutant *Ospair1 Osrec8 Ososd1* represents the *MiMe* genotype in rice that produces clonal gametes.

^b^ The column “Female parent construct” describes any parthenogenesis‐inducing element that has been used, which consists of a promoter and a parthenogenesis factor.

^c^ The most commonly used promoters are *EGG CELL 1.1* (*AtEC1.1*, AT1G76750) and *AtEC1.2* (AT2G21740, also referred to as *DOWNREGULATED IN DIF1 45* (*DD45*)).

^d^ The parthenogenesis‐inducing genes refer to their coding sequences, unless indicated otherwise (i.e., “geno” for genomic sequences); note that the lettuce *Lssex* gene is the ortholog of dandelion *PAR*.

^e^ The male parent describes the genotype of the pollen, which either is identical to the mother (selfing, indicated by *), or a haploid inducer line, or fluorescent marker line to facilitate selection of haploid progeny.

^f^ For allotetraploid Nicotiana (2*n* = 4*x* = 48), haploid indicates the presence of half the genome size of the maternal parent. These “haploid” ploidy levels are 1*n* = 2*x* = 24 when using *AtEC1.2*, and 2*n* = 4*x* = 48 when using *AtRDK2* due to spontaneous chromosome doubling.

A recent study by Chen et al. (2022) has explored the roles of *AtBBM* (*AtPLT4*) and its homolog (*AtPLT2*) in *Arabidopsis* seed development and applied the *Brassica napus BBM* (*BnBBM1*) gene to engineer parthenogenesis in *Arabidopsis*, *Brassica napus*, and tomato (Chen et al., 2022). In the zygote and endosperm, *AtBBM* and *AtPLT2* promote embryo progression and viability, as well as endosperm proliferation and cellularization, which is a critical developmental shift for embryo survival. In contrast, single mutants of these genes display accelerated embryo development, indicating that *AtBBM* and *AtPLT2* can also independently control the progression of embryo development. Additionally, this study shows that ectopic expression of *BnBBM1* in the egg cell can activate the development of haploid embryos in *Arabidopsis*, *Brassica napus*, and tomato, which could be used as a component of synthetic apomixis in those species. However, the ectopic expression constructs could not cause highly penetrant embryo induction, suggesting that finetuning of *BnBBM1* expression in seeds is necessary for successful parthenogenesis. Moreover, the diverse rates of abortion and aberrant embryo development of different constructs can be explained by the lack of endosperm development (Chen et al., 2022).

Interestingly, Chen et al. (2022) used *BnBBM1* rather than the native *AtBBM* gene to induce parthenogenesis in *Arabidopsis* to prevent potential gene silencing. Indeed, when aiming to express *AtBBM* in the *Arabidopsis* egg cell, expression was inhibited, thereby strongly reducing the occurrence of parthenogenesis (Liu, Han, et al., 2024). This inhibition was shown to be partially due to an RWP‐RK domain‐containing (RKD) transcription factor, AtRKD5, which recognizes the 3′ end of *AtBBM* and reduces *AtBBM* expression. To overcome this limitation, Liu, Han, et al. (2024) generated a codon‐optimized version of *AtBBM* and chimeric genes of the *Arabidopsis* and rice homologs, which increased the haploid induction rate up to 5% (Liu, Han, et al., 2024). Nevertheless, the *BnBBM1* gene still appears to be more functional for parthenogenesis in *Arabidopsis* and outperforms the engineered *BBM* versions (see also Table 1). Together, these studies suggest that context matters and that using native genes does not necessarily result in the strongest induction of parthenogenesis.

Building on earlier work in rice and maize (*Zea mays*) (Conner et al., 2017; Khanday et al., 2019) and the expression of *ZmBBM1* in maize egg cells have recently been shown to trigger the formation of haploid plants at high penetrance (65%) (Skinner et al., 2023). For this purpose, *ZmBBM1* was driven by the *Arabidopsis EGG CELL 1.2* (*AtEC1.2*) promoter (*AT2G21740*, also known as *DOWNREGULATED IN DIF1 45* (*AtDD45*)) (Skinner et al., 2023). In contrast to the findings in dicots (Chen et al., 2022; Liu, Han, et al., 2024), the studies in monocots indicate a benefit of utilizing native genes rather than foreign genes, potentially due to the interplay of BBM protein structure with target binding sequences, which may be a result of evolutionary constraint (Conner et al., 2017; Khanday et al., 2019; Skinner et al., 2023).

Related to the above approach in maize, Ye et al. (2024) developed a novel parthenogenetic double haploid approach by combining egg cell expression of *ZmBBM2* with *CYCLIN DELTA‐2* (*ZmCycd2*). Specifically, ectopic co‐expression of *ZmBBM2* and *ZmCycd2* in unfertilized egg cells via a *Panicum virgatum* egg cell promoter (*PvEC*) resulted in maternally derived diploid embryos. This *in vivo* approach, in conjunction with gene editing, allows for the creation of mature seeds from a maternally derived, gene‐edited diploid embryo without the need for colchicine‐based doubling or *in vitro* tissue culture. In conclusion, this is a novel method for producing gene‐edited maize double haploid populations with both natural and *de novo* phenotypic variation, which can expedite genetic gain per breeding cycle (Ye et al., 2024).

Prior research established rice parthenogenesis by using the *EC1.2* egg‐cell‐specific promoter to drive *OsBBM1* expression leading to a line that gives rise to 29% haploid progeny (Khanday et al., 2019). As *OsBBM1* is a paternally expressed gene it raised the possibility that additional paternally expressed genes could contribute to the initiation of embryogenesis following fertilization. Accordingly, Ren et al. (2024) found the WOX‐family transcription factor gene *DWARF TILLER1* (*OsDWT1*)/*WUSCHEL‐LIKE HOMEODOMAIN 9* (*OsWOX9A*) is paternally expressed in zygotes and is a strong enhancer of embryo initiation by *OsBBM1*. When those two genes are co‐expressed in egg cells, using the *EC1.2* egg‐cell‐specific promoter, a parthenogenesis rate of 86–91% results, representing a 4‐ to 15‐fold increase over *OsBBM1* alone (Ren et al., 2024). The increased frequencies of haploid progeny are stably propagated through multiple generations (Ren et al., 2024). However, egg‐cell‐specific expression of *OsWOX9A* alone showed no production of haploid progeny, indicating it is not a parthenogenetic factor itself (Ren et al., 2024). Additionally, twin rice plants arose from single seeds derived from plants that co‐express *OsBBM1 and OsWOX9A* in egg cells at a much greater rate than in previous reports, yet the origin of such twin seedlings remains unknown (Ren et al., 2024; Skinner et al., 2023; Vernet et al., 2022). Summarizing, *OsWOX9A* can operate as an enhancing factor of *OsBBM1* to increase the likelihood of establishing a zygotic state in rice (Ren et al., 2024).

The *ToPAR* gene cloned from apomictic dandelion is an alternative to *BBM* for parthenogenesis engineering. Large genetic deletions of the *LOSS OF PARTHENOGENESIS* (*LOP*) locus, and targeted CRISPR mutants of the *ToPAR* gene, in polyploid apomictic dandelion are sufficient for loss of apomixis and complete absence of viable seed production, the latter of which can be restored by pollination (Underwood et al., 2022). Only the dominant *ToPAR* allele is expressed in apomictic egg cells which is likely triggered by a miniature inverted‐repeat transposable element (MITE) insertion in the promoter (Underwood et al., 2022). Dandelion *LOP* mutants were complemented by driving the expression of the homologous lettuce (*Lactuca sativa*) gene, so‐called *Lssex*, with the MITE‐containing promoter indicating the regulatory sequence can act as a controlling element of reproductive mode. In addition, heterologous expression of *ToPAR* in lettuce egg cells leads to embryo‐like structures in the absence of fertilization (Underwood et al., 2022). In sexual species, *PAR* homologs are highly expressed in sperm cells, leading to a model where *PAR* transcripts and/or PAR proteins are delivered during fertilization to trigger embryogenesis. In contrast, in apomictic dandelion *PAR* is expressed in egg cells without fertilization, which causes cell division without gamete fusion (Underwood et al., 2022).

Beyond *Taraxacum*, the *PAR* gene appears to have been a target of convergent evolution of apomixis in two other Asteraceae genera: *Pilosella* and *Hieracium* (Bicknell et al., 2023; Underwood et al., 2022). In *Pilosella*, a *LOP* locus was independently identified and fully sequenced (Bicknell et al., 2023; Underwood et al., 2022). Surprisingly, the *Pilosella LOP* locus was syntenic to the dandelion locus and contained a *ToPAR* homolog that specifically on the dominant allele has a similar, yet different, MITE insertion in the promoter to that found in dandelion (Underwood et al., 2022). Extending these insights Bicknell et al. (2023) identified in apomictic *Hieracium*, a third insertion of a transposable element (TE) in the dominant *PAR* allele has occurred 154 bp upstream of the *PAR* start codon (Bicknell et al., 2023). The three transposon insertions are different in terms of length, location, terminal repeats, and internal sequences suggesting the existence of three separate ancestral alterations of the *PAR* locus (Bicknell et al., 2023; Underwood et al., 2022). Finally, the transfer of dominant wild‐type *Pilosella PAR* alleles through egg cells is only possible when they are transmitted together with a recessive sexual allele – haploid transfer was never detected through pollen or egg cells. While the functional role of dominant alleles is clear, the recessive ones may also play a role in *Pilosella* reproduction due to the requirement for their inheritance for successful reproduction (Bicknell et al., 2023).

In addition to the initial proof‐of‐principle of transferring *ToPAR* to lettuce (Underwood et al., 2022), it was recently demonstrated that the *ToPAR* gene from dandelion itself can surprisingly lead to parthenogenesis in monocot plants like foxtail millet and rice (Huang, Liang, et al., 2024; Song, Wang, Ji, et al., 2024). In foxtail millet, this was achieved by developing a vector expressing the *ToPAR* gene under the *AtEC1.2* promoter. In six of the 11 lines containing the vector haploid induction was observed at rates between 1.4% and 10.2%. Moreover, these plants exhibited a wide range of morphological variations including height and panicle length, while the seed setting rate significantly declined (Huang, Liang, et al., 2024). Overall, the studies in lettuce, foxtail millet, and rice lay an important foundation for future synthetic apomixis engineering using the *ToPAR* gene (Huang, Liang, et al., 2024; Song, Wang, Ji, et al., 2024; Underwood et al., 2022).

### Sporophytic apomixis

Complementary to the parthenogenetics factors, *BBM* and *PAR* are a class of *RWP* genes, encoding RWP‐RK domain‐containing proteins, which have been identified as factors involved in sporophytic apomixis in *Citrus*, *Fortunella*, and *Mangifera* (Nakano et al., 2012; Wang et al., 2017; Wang et al., 2022; Yadav et al., 2023). The *Citrus RWP* gene (*CitRWP*) was initially identified by a GWAS study of 45 polyembryonic apomicts and 63 monoembryonic fully sexual *Citrus* cultivars. A genetic marker of the *CitRWP* gene was further analyzed in 213 polyembryonic and 537 monoembryonic varieties which showed that the presence of a MITE insertion in the *CitRWP* gene promoter perfectly correlated with sporophytic apomixis in *Citrus* (Wang et al., 2017).

The genetic basis of sporophytic apomixis in the *Citrinae* has been further expanded by a recent study that developed genomic data and analyzed the presence of sporophytic apomixis in samples from the closely related *Poncirus*, *Fortunella*, and *Citrus* genera (Wang et al., 2022). This study demonstrated that apomixis is present in species from all three genera and concluded that introgression of a single *CitRWP* haplotype is not the main cause of apomixis across divergent *Citrus* and *Fortunella* accessions. Through the hybridization of sexual and apomictic *Fortunella hindsii* accessions, generation of a segregating population and QTL mapping, it was shown that the same locus controls apomixis in *Fortunella* as in *Citrus* (Wang et al., 2022). However, the *FhRWP* and *CitRWP* haplotypes are different: the *Fortunella* haplotype is 596 bp and contains three similar MITE insertions while the *Citrus* haplotype is 424 bp and contains two different MITE insertions. The genetic basis of apomixis in *Poncirus*, however, does not seem to be related to insertions in the *RWP* promoter. Overall, it appears that convergent evolution of apomixis has occurred within the *Citrinae*, much like in the Asteraceae genera *Taraxacum*, *Pilosella*, and *Hieracium*, and strikingly has relied on the insertion of TEs in five independent cases (Bicknell et al., 2023; Underwood et al., 2022; Wang et al., 2022).

Despite the pinpointing of the *CitRWP* in 2017 (Wang et al., 2017), only recently functional genetic validation was attempted in *Fortunella* (Song, Wang, Zhou, et al., 2024). Reduced expression level of *FhRWP* resulting from RNAi experiments led to decreased polyembryony and seed incidence (Song, Wang, Zhou, et al., 2024). However, CRISPR/Cas9‐based knockout of *FhRWP* led to growth and developmental defects that hindered blooming and fruit set, prevented seed production, and made it impossible to quantify polyembryony. In gain‐of‐function experiments constitutive expression of *FhRWP* was induced by agrobacterium‐mediated transformation of monoembryonic *Fortunella* epicotyl stem segments. This led to highly proliferative embryogenic callus, indicating the gene may facilitate somatic embryogenesis, yet no true transgenic plants could be regenerated (Song, Wang, Zhou, et al., 2024). In conclusion, the molecular genetic exploration of the *RWP* gene in *Fortunella* appears to be difficult due to pleiotropy and warrants further investigation.

On a mechanistic level, the insertion of a MITE transposon in the *FhRWP* promoter appears to increase chromatin accessibility at that locus and the role of other factors (FhARID and *Citrus sinensis* ZINC FINGER PROTEIN 7 (CsZFP7)) in polyembryony have been explored (Jia et al., 2023; Song, Wang, Zhou, et al., 2024). Through ATAC‐seq, more than 40 000 and 45 000 accessible chromatin regions in monoembryonic and polyembryonic ovules were identified, respectively (Song, Wang, Zhou, et al., 2024). This indicated the presence of the MITE in the apomictic allele appears to make the chromatin more accessible and the MITE is thought to be a FhARID1 binding site based on a previous yeast one‐hybrid screen (Song, Wang, Zhou, et al., 2024; Wang et al., 2022). Two other studies explored the role of C2H2 type zinc finger genes based on their expression patterns in polyembryonic and monoembryonic accessions and their possible role in nucellar embryogenesis (Jia et al., 2021; Jia et al., 2023). The knock down of one of these genes, *CsZFP7*, described as a homolog of dandelion *ToPAR*, increases the proportion of monoembryonic seeds in the T1 generation of mini‐citrus, even though not all the seeds became monoembryonic in the transgenic lines. This may be the result of the abundant expression of *CsZFP7* in polyembryonic ovules and the weak suppressive effect of RNAi transgene on *CsZFP7* expression (Jia et al., 2023). As a result, the upstream regulatory genes, such as *CitRWP*, which genetically controls polyembryony in citrus, may influence the expression of *CsZFP7* in polyembryony ovules (Wang et al., 2017). Summarizing, more research will hopefully resolve the molecular roles of *CitRWP*/*FhRWP*, *FhARID1*, and *CsZFP7* in polyembryony (Jia et al., 2023).

Outside of the *Citrinae*, a recent breakthrough in sporophytic apomixis was the fine‐mapping of the mango (*Mangifera indica*) locus for polyembryony and the further demonstration of convergent evolution of apomixis through a chloroplast DNA insertion in the promoter of the mango *CitRWP* homolog (*MiRWP*) (Yadav et al., 2023). Through the phenotyping of 93 polyembryonic accessions and 107 monoembryonic accessions, combined with sequence‐ and marker‐based genotyping, the mango polyembryony locus was mapped to a region containing only six predicted genes including *MiRWP*. Gene expression analysis in the early stages of seed fruit development demonstrated that *MiRWP* expression is higher in polyembryonic varieties compared with monoembryonic varieties. The authors propose that a chloroplast DNA insertion occurred after a whole genome duplication and show most of the polyembryonic accessions are heterozygous for the *MiRWP* allele, similar to the case in *Citrus*. This heterozygous state may be most common due to a substantial selection against homozygous genotypes (Yadav et al., 2023). In summary, in *Citrus*, *Fortunella*, and *Mangifera*, three independent promoter insertion events in a *RWP* gene promoter lead to the convergent evolution of nucellar embryogenesis and polyembryony (Wang et al., 2022; Yadav et al., 2023).

### Autonomous endosperm development

In most natural apomixis systems, endosperm formation is sexual (i.e., relying on the fertilization of the central cell by a sperm cell), however autonomous endosperm formation could be a useful trait in grass and bean species to ensure seed filling. Significant progress in this area has revolved around the *FERTILIZATION‐INDEPENDENT SEED* (*FIS*) class *Arabidopsis* mutants where an autonomous endosperm develops up to the stage of cellularization where it fails (Hands et al., 2016). These *FIS* class mutants are characterized by mutations in several *POLYCOMB REPRESSIVE COMPLEX 2* (*PRC2*) genes (e.g., *medea* (*mea*), *fertilization‐independent endosperm* (*fie*), *fis2*, and *multicopy suppressor of ira1* (*msi1*)) (Grossniklaus et al., 1998; Guitton et al., 2004; Kiyosue et al., 1999; Köhler et al., 2003; Luo et al., 2011; Ohad et al., 1999). More recently, active expression of *YUCCA 10* (*YUC10*), an auxin biosynthetic gene that is directly repressed by PRC2, in the central cell largely recreates the phenotype of the *FIS* class mutants – many divisions of the central cell but no true endosperm formation (Figueiredo et al., 2015).

A recent step toward autonomous endosperm engineering was the finding that expression of a usual sperm cell‐specific cyclin D gene (*AtCYCD7;1*) in the central cell led to the development of endosperm‐like structures (Simonini et al., 2024). The proliferation of the central cell is related to the degradation of RETINOBLASTOMA RELATED 1 (RBR1), which is an effective inhibitor of the progression of the S phase and G2 phase entry that is conserved through evolution (Avni et al., 2003; Knudsen et al., 2000; Weinberg, 1995). As a result, RBR1 is a key player in ensuring maternal and paternal genomes commence endosperm development in a synchronized manner (Johnston et al., 2008; Simonini et al., 2024). The main component in the RBR1 degradation upon fertilization is CYCD7;1, which is only found in the sperm nuclei of mature pollen (Simonini et al., 2024). Consistent with previous research (Sornay et al., 2015), central cell‐specific or ubiquitous ectopic expression of CYCD7;1 enhances the development of endosperm‐like structures in unfertilized ovules (Simonini et al., 2024). The RBR1‐CYCD7;1 interaction is mediated by a Leu‐x‐Cys‐x‐Glu motif (Lee et al., 1998; Matos et al., 2014), and CYCD7;1 variant lacking this motif are not capable of causing endosperm proliferation in unfertilized ovules, indicating that the RBR1‐CYCD7;1 interaction is essential for the progression of S phase in the central cell upon fertilization (Simonini et al., 2024). In summary, modulation of CYCD7;1 expression could be an important route to the development of autonomous endosperm.

Due to its function in the FIS‐PRC2 complex in *Arabidopsis*, mutation of *FIE* leads to autonomous initiation of endosperm development. Investigations into the rice homologs OsFIE1 and OsFIE2 however have led to some contradictory results. Studies by Li et al. (2014) and Cheng et al. (2020) found autonomous initiation of endosperm development in *OsFIE2*‐RNAi, *Osfie2* ^*+/−*^, and *Osfie1* ^*−/−*^ *Osfie2* ^*+/−*^ mutant genotypes, whereas a study by Nallamilli et al. (2013) did not find an autonomous endosperm phenotype for an *OsFIE2*‐RNAi line. A new study by Wu et al. (2023) revealed a novel function of OsFIE1 and OsFIE2 in repressing egg cell division in the embryo sac in the absence of fertilization. During this study, Wu et al. (2023) found that in addition to autonomous endosperm, the *Osfie1* ^*−/−*^ *Osfie2* ^*+/−*^ mutant also initiated asexual embryo formation. The formation of asexual embryos was also observed to a lower degree in the *Osfie2* ^*+/−*^ mutant but not in *Osfie1* single mutants. In this study, it was not clear if asexual embryos originated from the egg cell or synergid cells. However, the observance of degraded synergid cells, intact egg cells, and autonomous endosperm shortly after emasculation, together with the increase of asexual embryos at later timepoints, would suggest that the asexual embryos originate from the egg cell. For both the *Osfie* single mutants no autonomous endosperm was observed therefore suggesting that they act redundantly in repressing central cell division. Thus, this study indicates that the rice FIS‐PRC2 complex plays an important role in suppressing cell division in the embryo sac (Wu et al., 2023).

### Synthetic apomixis in rice

The development of synthetic apomixis systems in rice has rapidly advanced in recent years. This work was inspired by work on synthetic clonal seed production in *Arabidopsis* where clonal egg cells from a *MiMe* plant (*Atspo11 Atrec8 Atosd1*) were induced to form clonal embryos by maternal haploid inducer crosses with the *CENH3* tailswap line (Marimuthu et al., 2011). In rice, the combination of *MiMe* (*Ospair1 Osrec8 Ososd1*) and maternal haploid induction by the *Osmtl* mutant has also been shown to lead to clonal seeds (Wang et al., 2019). The application of haploid inducers like *CENH3* tailswap, *mtl/pla1/nld*, and *dmp* in synthetic apomixis studies usually leads to low penetrance clonal seed formation (Chen et al., 2024; Gilles et al., 2017; Kelliher et al., 2017; Liu et al., 2017; Marimuthu et al., 2011; Ravi & Chan, 2010; Wang et al., 2019; Zhong et al., 2019); therefore here we largely focus on combinations of *MiMe* with parthenogenesis in rice.

A recent major advance in synthetic apomixis combined the induction of *MiMe* mutations and egg cell expression of Os*BBM1* in a single construct in F1 hybrid rice (Vernet et al., 2022). This approach is similar to an earlier approach from Khanday et al. (2019), however by combining both traits on one construct, the efficiency of clonal seed production was increased from ~30% to up to 95% clonal seed production across multiple generations. No major differences in vegetative development were observed between apomictic lines and control F1 hybrid plants, although apomictic plants have decreased fertility. Interestingly, higher clonal seed rates seem to be associated with lower panicle fertility. This is exemplified by 80%–100% clonal seeds with a panicle fertility of 33%, in contrast to 55%–84% clonal seeds with a panicle fertility of 81% in another line (more examples are shown in Table 1). Furthermore, the consistent propagation of hybrid traits over two generations of clonal reproduction without obvious negative effects indicates that heterosis is predominantly genetically controlled in rice. This demonstrates that parental effects do not restrict the utility of synthetic apomixis for rice hybrids and possibly for other sexually reproducing crop plants. Taken together, this study showed that a high frequency of synthetic apomixis, stable across generations, in crop species is possible (Vernet et al., 2022).

Using a similar apomictic construct as Vernet et al. (2022), Song, Li, Chen, et al. (2024) also achieved high cloning efficiency and they went on to modify the system in an effort to increase the fertility of *MiMe‐OsBBM1* plants. In this research, two different promoters, the *EC1.2* from *Arabidopsis* and the *ECA1.1* from rice were used to drive *OsBBM1* expression and combined with *MiMe* gene mutagenesis to generate two apomixis inducing constructs (Song, Li, Chen, et al., 2024). As in previous studies, the authors found higher cloning efficiencies (more than 80% diploid offspring) that typically led to reduced fertility compared with wild‐type controls (Song, Li, Chen, et al., 2024). Subsequently, the apomixis systems were modified by introducing a SunTag gene activation system (Papikian et al., 2019) to enhance *OsBBM1* expression in the *MiMe‐EC1.1::OsBBM1* and *MiMe‐ECA1.1::OsBBM1* systems (Song, Li, Chen, et al., 2024). Following promising results with a small scale transformation of the enhanced *MiMe‐ECA1.1::OsBBM1* system (the so‐called “MiMe‐ECA1‐AZP2” system), the authors generated 307 fertile transgenic events. Within these plants, they identified 10 lines that gave rise to 95.3%–100.0% diploid progeny and exhibited seed setting rates between 80.7% and 86.7% (where wild‐type controls had 87.8% seed setting) (Song, Li, Chen, et al., 2024). The apomictic plants were investigated through the generations (T1–T3), showing a stable cloning rate and fertility (Song, Li, Chen, et al., 2024). This study demonstrates that the combination of enhancing *OsBBM1* expression and screening of a huge number of transgenic events can facilitate the selection of apomictic lines with superior fertility traits (Song, Li, Chen, et al., 2024).

Stability of plant phenotypes in clonal hybrids has also been addressed in another study up to the fourth transgenic generation (Liu et al., 2023). In this work, a previously described *Fix* system (*Ospair1 Osrec8 Ososd1 Osmtl*) combining *MiMe* and maternal haploid induction is exploited (Wang et al., 2019). In this *Fix* system, the clonal seed rate remained unaltered over multiple generations and was between 3.7% and 4.3% (Liu et al., 2023). Despite being low, the seed set of *Fix* plants (5.7%–7%) remained consistent across clonal generations compared with the control line (75.9%). Various other results supported the efficiency and stability of this apomixis system across generations including the genetically identical whole genomes and the relatively low proportion of differentially methylated regions between *Fix* and wild‐type plants across generations. Similarly, the transcriptome analysis showed that only 0.5% of the genes were differentially expressed between different clonal generations of *Fix* plants. In conclusion, *Fix* plants can stably clone themselves over multiple generations (Liu et al., 2023).

Several recent studies have concentrated on identifying and testing alternative parthenogenetic factors to *OsBBM1* for engineering synthetic apomixis in rice in combination with the *MiMe* system described above (Dan et al., 2024; Huang, Meng, et al., 2024; Song, Wang, Ji, et al., 2024; Wei et al., 2023). Wei et al. (2023) attempted to use *OsBBM2, OsBBM3, and OsBBM4* driven by *AtEC1.2*, for parthenogenesis engineering in hybrid rice. Transgenic plants containing the respective genes have similar vegetative development and morphology, but only *OsBBM4* was able to induce parthenogenesis and trigger haploids in hybrid rice at a haploid induction rate of 3.2%. In addition, the combination of *OsBBM4* egg cell expression with the *MiMe* system in so‐called “*Fix2* plants” led to clonal reproduction at a rate of 1.7% in hybrid rice, while still maintaining a high seed setting rate that was comparable to wild‐type controls (Wei et al., 2023).

In contrast, Mengqui Song *et al*. (2024) heterologously expressed *ToPAR* in combination with the rice *MiMe* system, resulting in synthetic apomixis at a maximal cloning rate of 67%. This study represented the first demonstration that the dandelion ToPAR protein could function in a monocot species – a remarkable finding given the monocotyledonous and dicotyledonous plants diverged more than 200 million years ago. Rice *MiMe‐ToPAR* plants did not have any significant differences from wild‐type controls in terms of physiology and seed set (Song, Wang, Ji, et al., 2024). Also, consistent with the study of Wei et al. (2023), the heterozygosity was retained, and clonal propagation was fixed indicating this system can also cause highly fertile synthetic apomixis in hybrid rice, supporting the propagation of hybrids. Additionally in this study, the synthetic apomixis approach is also combined with improved agronomic traits through the mutation of genes relating to brittle culm and semi‐dwarfism, further demonstrating the possibilities of biotechnological apomixis breeding (Song, Wang, Ji, et al., 2024).

In another study, the rice *MiMe* system was combined with the expression of endogenous rice gene *OsWUS* driven by the *AtEC1.2* promoter, leading to a maximal cloning rate of 22% in hybrid rice (Huang, Meng, et al., 2024). The seed setting rate of the so‐called “*Fix3* plants” (80.8%) was as high as wild‐type controls (80.1%). The “*Fix3* plants” have normal development and vegetative morphology, and at partial penetrance, egg cell fertilization is not required due to ectopic expression of *OsWUS*. Similarly to the previous studies, the above characteristics propagated through generations while the heterozygosity was maintained suggesting that hybrid vigor, including high fertility was heritable (Huang, Meng, et al., 2024). Finally, Dan et al. (2024) combined the *MiMe* system with *OsBBM1*, expressed under the *AtEC1.2* promoter, and with *AtWUS*, expressed under the *OsMYB98* and *OsECA1* promoters, resulting in the production of high‐efficacy clonal seeds in hybrid rice. However, the fertility of those two lines varied significantly, with the highest seed setting of those being comparable with the wild‐type (Dan et al., 2024). Moreover, the clonal seed rate was higher in the *OsBBM1*‐*MiMe* construct compared with *OsBBM1‐* and *AtWUS‐*containing ones (Dan et al., 2024). Summarizing, since the Khanday et al. (2019) study, several alternatives to *OsBBM1*, including *OsBBM4*, *ToPAR*, and *OsWUS* have been successfully shown to be compatible with *MiMe* to engineer synthetic apomixis in rice.

## BOTTLENECKS FOR ENGINEERING SYNTHETIC APOMIXIS

**Figure 3:**
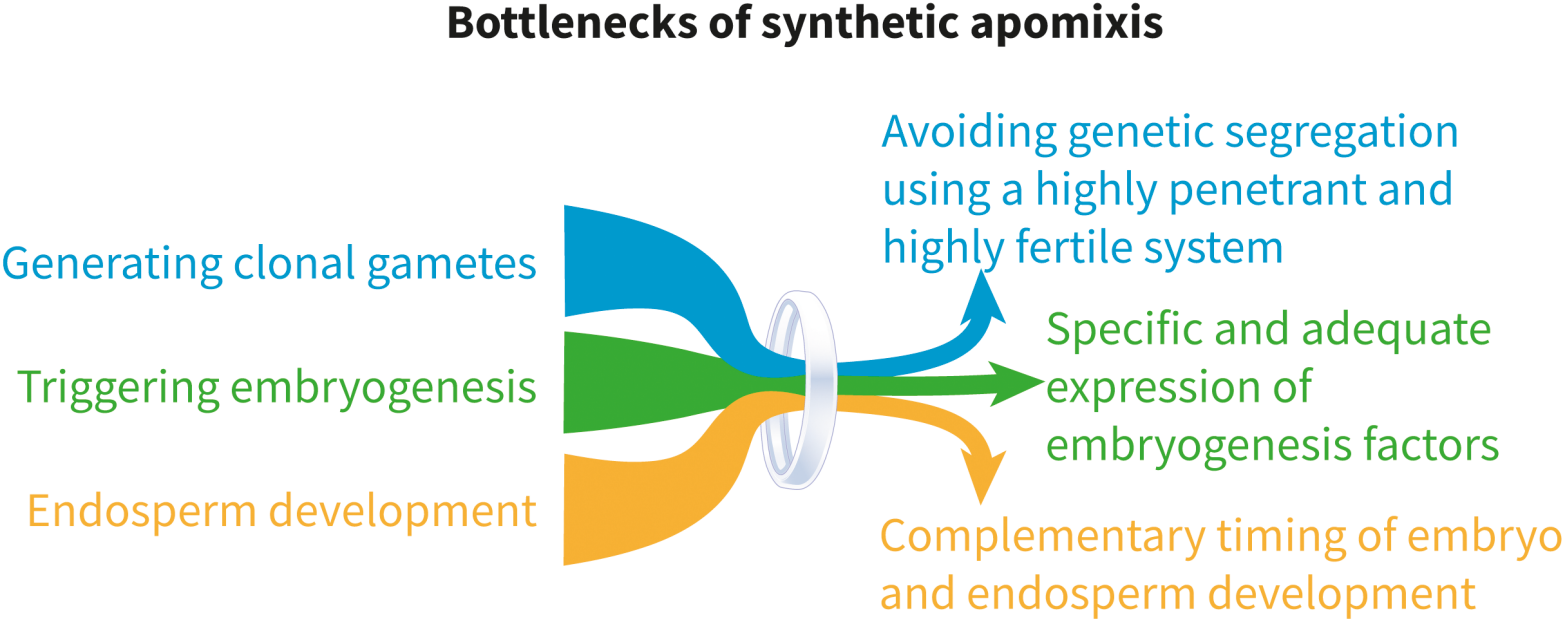
Bottlenecks of synthetic apomixis. Schematic representation of the major bottlenecks of clonal seed production through synthetic apomixis.

Despite the recent advances in our understanding of plant reproduction, there are still several bottlenecks in synthetic apomixis to generate clonal seeds in crop species. These bottlenecks reside in the consecutive steps of apomixis, being altered meiosis, parthenogenesis, and endosperm development. An overview of major bottlenecks in engineering synthetic apomixis are shown in Figure 3, and a related set of open questions are listed in Box 2.

Box 2 **Open questions**

- What is the genetic basis of apomeiosis and autonomous endosperm in naturally apomictic plants?
- Is there a species‐universal highly penetrant mutation, which abolishes the second meiotic division?
- Which gene, or combination of genes, has the greatest potential to induce parthenogenesis, in any given species?
- How can suitable spatiotemporal expression of parthenogenesis genes be engineered by design?
- Is autonomous endosperm development necessary for high‐penetrance, high‐fertility synthetic apomixis?

### Skipping meiosis

The first step in synthetic apomixis is the production of clonal gametes. Until today, no natural apomeiosis gene has been identified and successfully transferred to another plant species. This is therefore a major bottleneck and there is a need for the genetic identification and functional validation of natural apomeiosis genes which can be applied in crop species.

#### How to translate the MiMe genotype to a wide variety of crops?

Currently, clonal gametes are usually engineered through *MiMe*, which requires the modification of multiple genes to alter different aspects of meiosis. The *MiMe* system has only been set up in a limited number of species – *Arabidopsis*, rice and tomato – but still needs to be explored in other angiosperms (Cifuentes et al., 2016; D'Erfurth et al., 2009; D'Erfurth et al., 2010; Khanday et al., 2019; Mieulet et al., 2016; Wang et al., 2019; Wang et al., 2024). Due to different evolutionary trajectories and thereby variable histories of polyploidy, the process of establishing *MiMe* in different crops is quite complex and time‐consuming, as it is likely for each crop different mutant combinations must be tested. This difficulty is illustrated by the attempt to engineer *MiMe* in cotton (*Gossypium hirsutum*), in which the selected mutations all led to infertility (Qian et al., 2024). Alternatives to *MiMe*, although less applicable still, do exist and have been reviewed by Underwood and Mercier (2022).

To translate the existing *MiMe* systems to other species, an expansion of our toolbox of meiotic cell‐cycle control genes is required. A large number of genes involved in meiosis in plants have been identified (Mercier et al., 2015). The genes playing a role in the first two building blocks of *MiMe* (abolishing meiotic recombination and separating the sister chromatids) are strongly conserved (Underwood & Mercier, 2022); therefore, identification of homologous genes is feasible. The bottleneck is the third building block, the skipping of the second meiotic division, which is crucial to generate unreduced gametes, and novel mutants that skip this process are of high interest. It is important that this step is engineered with a high penetrance. Mutations in the first two building blocks only (i.e., in *SPO11‐1* and *REC8*) are lethal due to unbalanced distributions of chromosomes, thus, viable gametes can only be obtained if also the second meiotic division is omitted (D'Erfurth et al., 2009).

In *Arabidopsis*, skipping meiosis II is most effective by mutating *AtOSD1*, having a higher penetrance than mutating its alternatives *THREE‐DIVISION MUTANT 1* (*AtTDM1*) or *AtTAM* (Cifuentes et al., 2016; D'Erfurth et al., 2009; D'Erfurth et al., 2010). For other species than *Arabidopsis*, this step is a bottleneck, as exemplified in tomato and watermelon (*Citrullus lanatus*). Importantly, *AtOSD1* acts partially redundantly with its paralog *UV‐B‐INSENSITIVE 4* (*AtUVI4*) in *Arabidopsis* and the double mutant *Atosd1 Atuvi4* displays aberrant nuclear divisions during gametogenesis, leading to embryo lethality (Iwata et al., 2011). However, many species, including tomato and watermelon, have a single, likely essential *OSD1*‐like gene. Only the *Brassicaceae* and few specific other species (including soybean, cassava, rice, and maize) have more than one *OSD1*‐like genes arisen from independent duplication events (Mieulet et al., 2016; Pang et al., 2024). In tomato, no viable null diploid *Slosd1* mutants could be obtained (Di et al., 2022; Wang et al., 2024). Instead, the partially penetrant *Sltam* mutation was used, resulting in *MiMe* plants with reduced seed set (Wang et al., 2024). In diploid watermelon, ClOSD1 likely functions not only in meiosis, but also in mitosis of somatic cells (Pang et al., 2024). The watermelon *Closd1* mutants are proposed to undergo somatic genome doubling as all regenerated *Closd1* mutants were tetraploid and no null diploids were found (Pang et al., 2024). Despite this *Closd1* mutants do skip the second meiotic division and thus their gametes are tetraploid (Pang et al., 2024). These examples illustrate that skipping meiosis II through *OSD1* gene mutation is not straightforward in multiple crops. Thus, for most crops, a highly penetrant yet viable and specific mutation to abort the second meiotic division is still lacking and for each new crop system, the meiotic cell‐cycle control needs to be investigated.

Highly penetrant *MiMe* mutations are desired to obtain fertile *MiMe* plants. In *Arabidopsis*, a fully penetrant apomeiosis phenotype can be induced, resulting in a viability of pollen and ovules similar to wild‐type levels (D'Erfurth et al., 2009). In contrast, lower penetrance may lead to an increased proportion of gametes with unbalanced chromosome distributions, thereby reducing fertility. Such reduced fertility is reflected in lower pollen viability, which reflects male fertility only, or in reduced seed set, due to reduced male and/or female fertility. This has been exemplified by tomato *MiMe* plants, in which both pollen viability and seed set were decreased due to incomplete penetrance of the *MiMe* genotype (Wang et al., 2024). Partial penetrance of a *MiMe* system could also reduce seed set due to ploidy mismatches between the pollen and endosperm (Mieulet et al., 2016), which is another bottleneck discussed below.

### Parthenogenesis

The second step in apomixis is the induction of the egg cell development into an embryo without fertilization by parthenogenesis. Despite progress with *BBM* (Chen et al., 2022; Dan et al., 2024; Qi et al., 2023; Ren et al., 2024; Song, Li, Chen, et al., 2024; Vernet et al., 2022; Wei et al., 2023; Ye et al., 2024; Zhang et al., 2020), *PAR* (Huang, Liang, et al., 2024; Song, Wang, Ji, et al., 2024; Underwood et al., 2022), and *WUS* (Dan et al., 2024; Huang, Meng, et al., 2024), significant bottlenecks must be overcome to establish fully penetrant synthetic apomixis in both dicot and monocot crop species. Since synthetic parthenogenesis is a relatively recent innovation and the difficulties in studying the female gametophyte due to its embedded position within the ovule, there is little knowledge on the molecular basis of synthetic parthenogenesis, so here we speculate about the potential bottlenecks.

#### Which gene(s) in which crop?

Determining the suitable parthenogenic factor or combination of factors for individual crops remains a bottleneck. In all crops, high penetrance of clonal reproduction is required as a mixture of sexual and clonal seeds is not desirable as seed lots will be a mixture of clonal and recombinant offspring. In addition, for grain crops, high fertility and grain filling is required as this is the final product on the farm, whereas in vegetable crops, this is of relatively minor significance.

By far the greatest progress in synthetic apomixis has been made in rice using *OsBBM1*, *ToPAR*, or *OsWUS* which have been reported to have maximal clonal rates of >95%, 67%, and 22% respectively (Table 1) (Dan et al., 2024; Huang, Meng, et al., 2024; Song, Li, Chen, et al., 2024; Song, Wang, Ji, et al., 2024; Vernet et al., 2022). Despite these impressive clonal rates, typically, lower fertility is found which represents a strong bottleneck for engineering synthetic apomixis since high efficiency and a good yield will be required on the field. The reports of these two newly discovered parthenogenic factors (*ToPAR* and *OsWUS*) now provide the opportunity to express multiple factors at once in the egg cell (Huang, Liang, et al., 2024; Underwood et al., 2022). Even though, for now, only single parthenogenic factors have been reported in apomictic plants, those apomictic species might have also evolved optimizations (i.e., epistatic interactions) in their embryogenic pathways to make those individual factors more efficient and thereby not require additional paternal factors (RNA and/or proteins delivered by the sperm cell, or genes expressed from the paternal genome in the zygote) for embryogenesis to occur (Conner et al., 2015; Underwood et al., 2022). Therefore, to convert sexual plants into apomicts multiple paternal factors might be required to induce efficient parthenogenesis.

Even though few paternal factors have been identified to date, the potential importance of them is exemplified by *OsWOX9A* in rice (Ren et al., 2024), as has been extensively described in the section Synthetic apomixis in rice. The combination of co‐expressing *OsWOX9A* together with *OsBBM1* to increase parthenogenesis by 4–15‐fold is a classical example of genetic enhancement (Ren et al., 2024). Other factors that could be important paternal factors are *SHORT SUSPENSOR* (*AtSSP*) and the *PAR* homologs *DUO1‐ACTIVATED ZINC FINGER 3* (*AtDAZ3*) and *TRANSCRIPTIONAL REPRESSOR OF EIN3‐DEPENDENT EHTYLENE‐RESPONSE 1* (*AtTREE1*) which have been reported to be paternally derived in Arabidopsis (Bayer et al., 2009; Cheng et al., 2024; Wang et al., 2021). At*SSP* is expressed in the pollen but only gets translated once the sperm cell has fused with the egg cell. If *AtSSP* is absent, zygote patterning is not properly established which subsequently results in a malformed suspensor (Bayer et al., 2009; Wang et al., 2021). Similarly, *AtDAZ3* AND *AtTREE1* in the zygote are sperm cell‐derived and double mutants of both genes lead to altered cell division patterns in early‐globular‐stage embryos (Cheng et al., 2024).

The identification of more natural parthenogenesis factors will increase the potential for engineering efficient and fertile synthetic parthenogenesis in diverse crop plants. Since the genes responsible for parthenogenesis in *Pennisetum* and *Taraxacum* differ, it is reasonable to suspect that apomictic species in the genera *Boechera, Rubus, Erigeron*, and others might also possess novel parthenogenesis genes, thus providing opportunities for identifying novel parthenogenic factors (Conner et al., 2015; Underwood et al., 2022). In addition to new parthenogenesis factors, sporophytic apomixis genes responsible for nucellar embryony like *RWP* from *Citrus* and *Mangifera* hold promise (Wang et al., 2022; Yadav et al., 2023). Since these genes trigger spontaneous embryo formation from nucellar tissue, they may also have the capacity to initiate parthenogenesis if expressed in egg cells.

#### Which promoter is most suitable to drive parthenogenesis genes?

Since parthenogenic efficiency represents a strong bottleneck, it is essential to consider also the regulatory elements that are being used to express parthenogenic factors. Partially penetrant clonal reproduction means that in some egg cells, the parthenogenic factor is not able to trigger parthenogenesis, therefore suggesting either unsuitable expression level (either too high or too low) of the parthenogenic factor, mistiming of expression or expression in the wrong cell type. Ideally, the promoter used will have egg cell‐specific expression and sufficient expression to induce parthenogenesis. It is possible that expression levels that are too high trigger uncontrolled cell division (Conner et al., 2015; Underwood et al., 2022). To achieve egg cell‐specific expression most current studies have employed the *Arabidopsis EC1.1* or *EC1.2* promoters. However, they may not be optimal and further promoter discovery could identify more suitable promoters of parthenogenesis engineering. This is supported by results of Song, Li, Chen, et al. (2024) whom observed an improvement in apomictic offspring after utilizing the SunTag gene activation system to enhance the expression of the rice ECA1.1 promoter (Song, Li, Chen, et al., 2024).

#### How to engineer parthenogenesis without genetic modification?

Besides biological constraints regarding the efficacy of the selected promoters and genes, the requirement for a GMO approach and the regulation associated therewith is another bottleneck in the application of parthenogenesis and synthetic apomixis in agriculture. The current induction of parthenogenesis is achieved by introducing an expression cassette for egg cell‐directed expression of one or multiple parthenogenesis factor(s) (Table 1). This thus requires a GMO approach, potentially making such parthenogenesis systems less attractive for breeding organizations due to the prohibitive GMO regulatory processes in many countries worldwide.

A solution for engineering GMO‐free parthenogenic plants may be found in the natural properties of TEs. TEs are important drivers of evolution and naturally change their location in the genome, thereby activating or repressing nearby genes (Castanera et al., 2023). A major difference between sexual and asexual reproductive modes in *Taraxacum*, *Pilosella*, *Hieracium*, *Fortunella*, and *Citrus* species is the insertion of TEs/repetitive elements in their apomixis gene promoters (Nakano et al., 2012; Underwood et al., 2022; Wang et al., 2022). Such insertions are thought to cause expression in female reproductive cells. Often TEs are epigenetically silenced and it will be enlightening to understand whether these TE insertions exert control through purely genetic or a combination of genetic and epigenetic mechanisms. Thus, the complete regulatory control of *PAR* and *RWP* alleles has not been completely unraveled yet, but it illustrates that in nature, TE insertions can act as controlling elements of genes involved in reproductive mode (McClintock, 1956; Nakano et al., 2012; Underwood et al., 2022; Wang et al., 2022).

Activated transposition of TEs could be used to integrate TEs in the promoter of *PAR, RWP*, or homologous genes, either randomly (GMO‐free) or in a targeted manner (by gene editing). In a GMO‐free approach, the increased mobilization of TEs under stress conditions could be used. Under normal conditions, TEs are under epigenetic control to silence the expression of TE‐encoded genes required for transposition (Nozawa et al., 2021). The transposition of TEs is inhibited by a plethora of inter‐linked silencing pathways which are initiated by RNA‐directed DNA methylation (RdDM) (Matzke & Mosher, 2014). RdDM, involving to different extents the RNA Polymerases II, IV, and V and a plethora of other factors, may be reduced in some stress situations which can lead to TE mobilization (Matzke & Mosher, 2014). Retrotransposons, the largest class of TEs in plants, can be activated and mobilized by heat stress, an RNA Pol II inhibitor (α‐amanitin), and a DNA methyltransferase (zebularine) in *Arabidopsis* and rice, leading to higher copy numbers of TEs (Thieme et al., 2017). This combination allows for increased TE activity, which could potentially be utilized in large forward genetic screenings to find TE insertions near apomixis genes leading to ectopic expression, thereby inducing embryo development without fertilization in a potentially GMO‐free fashion.

By using gene editing, complete promoters or only a specific TE can be inserted in a sequence‐specific target site in the genome. Several approaches for this have been developed, of which the recent transposase‐assisted target site integration (TATSI) method shows the highest integration efficiency (Liu, Panda, et al., 2024). This system combines the specificity of CRISPR‐Cas targeting with the seamless integration of TEs in the genome to insert sequences at specific genomic locations. In short, the donor sequence needs to be flanked by an *mPing* element, which is recognized for excision from the genome by the *Pong* transposase and then directed to a specific genomic location for integration by a gRNA (Liu, Panda, et al., 2024). Potentially, this TATSI system can be used to insert a TE or other egg cell‐specific elements in the promoter of an apomixis gene thereby activating expression in female reproductive cells.

### Nourishing the embryo

Autonomous endosperm development might offer some significant advantages over sexual endosperm (produced by pseudogamy) for engineering apomictic crops and especially for seed crops, as it avoids the problem of complementary timing/fertilization of embryo and endosperm development.

#### Sexual endosperm – Is it a problem?

Current studies on synthetic apomixis rely on sexual endosperm development for seed set. However, in many of these studies, the seed set of the synthetic apomictic crops has been found to be variable. In some studies, the seed set is close to wild‐type, but in others, seed set of the synthetic apomictic crops is significantly reduced (Dan et al., 2024; Liu, Wang, et al., 2024; Song, Wang, Ji, et al., 2024; Vernet et al., 2022; Wang et al., 2019; Wei et al., 2023). Due to the *MiMe* genotype generating unreduced male and female gametes, the ploidy of the endosperm will be modified. For example, in the rice *MiMe* system, the 4n central cell is fertilized by a 2n sperm cell to give rise to a hexaploid endosperm that maintains the 2:1 maternal‐to‐paternal ratio which is important for endosperm development (Mieulet et al., 2016). Despite the hexaploid endosperm seemingly not being a major bottleneck in rice – as several studies have successfully introduced *MiMe* in rice with high seed set – it might explain the variation in seed set between studies and could prove to be a bottleneck when introducing *MiMe* into other crops. As pointed out by Vernet et al. (2022), the introduction of parthenogenesis might block the fertilization of the central cell because fertilization depends on the secretion of EC1 proteins by the egg cell (Sprunck et al., 2012). Therefore, early initiation of parthenogenesis might block fertilization of the central cell if not enough EC1 proteins are present. In the study of Vernet et al. (2022), this seems not to be a major problem since their *MiMe* and *MiMe*‐*OsBBM1* lines have a similar seed set. Despite this, the synchronization of egg cell parthenogenesis and central cell fertilization could prove to be a bottleneck when engineering apomixis in other crops.

#### Autonomous endosperm – How to make it?

Autonomous endosperm might solve the potential issues above associated with pseudogamy if embryo and endosperm development can be triggered simultaneously at complete penetrance. However, the evolution of autonomous endosperm is relatively rare in apomictic plant species (Noyes, 2007). This suggests that either it is a complex trait to evolve, or the negatives outweigh the benefits for most apomictic species. In the few species like dandelion and hawkweed that did acquire this trait, there is little knowledge on the loci that are responsible for it (Rojek & Ohad, 2023; Van Dijk et al., 2020). Nevertheless, investigation into factors that control endosperm development in non‐apomictic species has provided some insights that might eventually lead to synthetic engineering of autonomous endosperm development. These factors include the FIS‐PRC2 complex, RBR1, CYCD7, and YUC10; and these have been discussed above in the recent advances (Chaudhury et al., 1997; Figueiredo et al., 2015; Grossniklaus et al., 1998; Kiyosue et al., 1999; Köhler et al., 2003; Ohad et al., 1996; Simonini et al., 2024; Wu et al., 2023). However, modulation of these factors fail to lead to complete endosperm development possibly due to an imbalance in the 2:1 maternal‐to‐paternal ratio, and are therefore likely not enough to engineer autonomous endosperm in crops. More knowledge on the downstream factors that are modified by these mutants will provide more insights on why development fails. In addition, further understanding of the bi‐directional signaling between endosperm development and seed coat development may be a further source of leads for engineering autonomous endosperm. Finally, exploring the molecular basis of autonomous endosperm in apomictic species like dandelion and *Hieracium* holds opportunities to find new factors responsible for endosperm development. In both these species, the autonomous endosperm trait is not linked together with apomeiosis and parthenogenesis (Henderson et al., 2017; Van Dijk et al., 2020).

Besides overcoming possible fertility issues related to synthetic apomixis, autonomous endosperm might provide other benefits when introduced into crop species. If autonomous endosperm is used in the context of synthetic apomixis, it effectively abandons the need for fertilization and thus creates plants that are completely autonomous. This is particularly interesting in crops that are grown for fruits and seeds, in which the development of the male gametophyte is significantly affected by heat stress. Often in these crops heat waves can lead to a significant lowering of pollen viability that subsequently results in unsuccessful pollination and therefore leads to yield loses. The non‐reliance of autonomous apomicts on pollen would allow the plants to set seed and thus fruit under heat stress conditions. Autonomous apomictic crops could also reduce some of the concerns with potential gene flow into wild relatives of GM‐crops. If these GM‐crops would reproduce completely autonomously; male sterility could be introduced and thus eliminating the risk of outcrossing to wild relatives through pollen.

## THE POTENTIAL OF CLONAL SEEDS

Apomixis is considered the holy grail in plant breeding because it facilitates the generation of clonal seeds and thereby the stable inheritance of hybrid vigor (Jefferson, 1994; Spillane et al., 2004; Underwood & Mercier, 2022). In this review, we have highlighted the recent advances in the understanding and engineering of apomixis and elaborated on the current bottlenecks of generating clonal seeds. The above bottlenecks all affect plant fertility, which is the major limiting factor in synthetic apomixis. So far, impressive progress has been made in generating clonal rice grains. In rice and other seed crops, both a high percentage of clonal seeds and a high seed set is desired, but generally, high clonal seed rates are associated with decreased fertility (see Table 1). As fertility directly relates to seed set and thereby grain yield, even marginally reduced fertility is a major bottle neck to agricultural application in grain crops.

Synthetic apomicts in rice have been obtained by combining the *Ospair1 Osrec8 Ososd1* mutations with ectopic expression of a *BBM*‐like gene (Dan et al., 2024; Khanday et al., 2019; Vernet et al., 2022; Wei et al., 2023), *ToPAR* (Song, Wang, Ji, et al., 2024), or *OsWUS* (Huang, Meng, et al., 2024). Up to 95% of clonal offspring can be obtained, but the seed set of these plants is often reduced and highly variable (15%–88%) (Dan et al., 2024; Vernet et al., 2022), although the recent report of Song, Li, Chen, et al. (2024) indicates there may be ways to overcome this (Song, Li, Chen, et al., 2024). In contrast to grain crops, in fruit and vegetable crops, high seed set is less essential because it does not (directly) influence crop yield. Although synthetic apomixis has not yet been demonstrated in these crops, its building blocks *MiMe* and parthenogenesis have been established in tomato (Chen et al., 2022; Wang et al., 2024). Thus, while reduced fertility of synthetic apomicts is still a major concern in grain crops, this approach offers great potential for fruit and vegetable crops, in which synthetic apomixis has not yet been fully explored.

As such apomixis in fruit and vegetable crops is a promising field for future research. First, a functional *MiMe* system in the important vegetable/fruit crop tomato has been developed and despite fertility issues, they are of relatively less importance compared with grain crops. Second, apomixis may facilitate the fixing of genotypes in species with high genomic heterozygosity, such as potato. Potato cultivars are classically tetraploid and obligatory outcrossing; thus, favorable genotypes cannot be fixed in seeds and are generally clonally propagated by tubers. While it is possible to inbreed diploid potatoes toward the generation of stable diploid parental lines, this process is highly complex due to inbreeding depression and the generation of fully homozygous elite parents has proved difficult (Zhang et al., 2021). Therefore, the introduction of apomixis in potato, and other crops with heterozygous genomes, may be an alternative to generate stable, high‐performing, hybrid genotypes through clonal seeds.

The most exciting prospect of clonal seed production is the fixing of hybrid genotypes, especially of those displaying hybrid vigor, as frequently mentioned in synthetic apomixis studies (a.o. (Huang, Meng, et al., 2024; Khanday et al., 2019; Underwood et al., 2022; Vernet et al., 2022)). On top of increases in plant performance, clonal seed production will ease the generation of large quantities of hybrid seeds from such high‐performing genotypes. This would allow for increased variation in the current monoculture system, with more varieties per crop, thereby increasing the robustness of agricultural systems (Dijk et al., 2016). To conclude, the improvement of crops by humans has taken place since the dawn of modern civilization and must continue if we as a global community are to overcome the problems of high disease pressure and climate change. A natural extension of thousands of years of man‐made selections is to harness modern technologies including genomic selection, genome engineering and synthetic apomixis to expedite the improvement of hybrid crops to infinity and beyond.

## CONFLICT OF INTEREST STATEMENT

CJU is listed as an inventor on two patent applications related to the contents of this review: “Gene for Parthenogenesis”, WO2020239984, owned by KeyGene N.V.; “Unreduced Clonal Gamete Formation and Polyploid Genome Design in the Solanaceae”, WO2024256682, owned by the Max‐Planck‐Gesellschaft.

## Data Availability

Data sharing is not applicable to this article as no new data were created or analyzed in this study.

## References

[tpj70054-bib-0001] Avni, D. , Yang, H. , Martelli, F. , Hofmann, F. , ElShamy, W.M. , Ganesan, S. et al. (2003) Active localization of the retinoblastoma protein in chromatin and its Response to S phase DNA damage. Molecular Cell, 12, 735–746. 14527418 10.1016/s1097-2765(03)00355-1

[tpj70054-bib-0002] Awan, M.J.A. , Amin, I. , Hensel, G. & Mansoor, S. (2024) Clonal gamete‐mediated polyploid genome design for stacking genomes. Trends in Plant Science, 29, 1285–1287. 39097426 10.1016/j.tplants.2024.07.010

[tpj70054-bib-0003] Bayer, M. , Nawy, T. , Giglione, C. , Galli, M. , Meinnel, T. & Lukowitz, W. (2009) Paternal control of embryonic patterning in Arabidopsis thaliana. Science, 323, 1485–1488. 19286558 10.1126/science.1167784

[tpj70054-bib-0004] Bicknell, R. , Gaillard, M. , Catanach, A. , McGee, R. , Erasmuson, S. , Fulton, B. et al. (2023) Genetic mapping of the LOSS OF PARTHENOGENESIS locus in Pilosella piloselloides and the evolution of apomixis in the Lactuceae. Frontiers in Plant Science, 14, 1239191. Available from: 10.3389/fpls.2023.1239191 37692427 PMC10485273

[tpj70054-bib-0005] Boutilier, K. , Offringa, R. , Sharma, V.K. , Kieft, H. , Ouellet, T. , Zhang, L. et al. (2002) Ectopic expression of BABY BOOM triggers a conversion from vegetative to embryonic growth. Plant Cell, 14, 1737–1749. 12172019 10.1105/tpc.001941 PMC151462

[tpj70054-bib-0006] Castanera, R. , Morales‐Díaz, N. , Gupta, S. , Purugganan, M. & Casacuberta, J.M. (2023) Transposons are important contributors to gene expression variability under selection in rice populations. eLife, 12, RP86324. 37467142 10.7554/eLife.86324 PMC10393045

[tpj70054-bib-0007] Chaudhury, A.M. , Ming, L. , Miller, C. , Craig, S. , Dennis, E.S. & Peacock, W.J. (1997) Fertilization‐independent seed development in Arabidopsis thaliana. Proceedings of the National Academy of Sciences, 94, 4223–4228. 10.1073/pnas.94.8.4223 PMC20611 9108133

[tpj70054-bib-0008] Chen, B. , Maas, L. , Figueiredo, D. , Zhong, Y. , Reis, R. , Li, M. et al. (2022) BABY BOOM regulates early embryo and endosperm development. Proceedings of the National Academy of Sciences, 119, e2201761119. 10.1073/pnas.2201761119 PMC9231476 35709319

[tpj70054-bib-0009] Chen, W.‐Q. , Xu, L. , Rao, Y. , Liu, C. , Hong, Z. , Lu, H. et al. (2024) Self‐propagated clonal seed production in dicotyledonous Arabidopsis. Science Bulletin, S2095‐9273(24)00886‐7. Online ahead of print. 10.1016/j.scib.2024.12.003 39672710

[tpj70054-bib-0010] Cheng, T. , Liu, Z. , Li, H. , Huang, X. , Wang, W. , Shi, C. et al. (2024) Sperm‐origin paternal effects on root stem cell niche differentiation. Nature, 634, 220–227. 39198649 10.1038/s41586-024-07885-0

[tpj70054-bib-0011] Cheng, X. , Pan, M. , E, Z. , Zhou, Y. , Niu, B. & Chen, C. (2020) Functional divergence of two duplicated fertilization independent endosperm genes in rice with respect to seed development. The Plant Journal, 104, 124–137. 33463824 10.1111/tpj.14911

[tpj70054-bib-0012] Cifuentes, M. , Jolivet, S. , Cromer, L. , Harashima, H. , Bulankova, P. , Renne, C. et al. (2016) TDM1 regulation determines the number of meiotic divisions. PLoS Genetics, 12, e1005856. 26871453 10.1371/journal.pgen.1005856 PMC4752240

[tpj70054-bib-0013] Conner, J.A. , Mookkan, M. , Huo, H. , Chae, K. & Ozias‐Akins, P. (2015) A parthenogenesis gene of apomict origin elicits embryo formation from unfertilized eggs in a sexual plant. Proceedings of the National Academy of Sciences of the United States of America, 112, 11205–11210. 26305939 10.1073/pnas.1505856112 PMC4568661

[tpj70054-bib-0014] Conner, J.A. , Podio, M. & Ozias‐Akins, P. (2017) Haploid embryo production in rice and maize induced by PsASGR‐BBML transgenes. Plant Reproduction, 30, 41–52. 28238020 10.1007/s00497-017-0298-x

[tpj70054-bib-0015] Cornaro, L. , Banfi, C. , Cavalleri, A. , Dijk, P.J. , Radoeva, T. , Cucinotta, M. et al. (2024) Apomixis at a high resolution: unravelling diplospory in Asteraceae. Journal of Experimental Botany, erae477. Online ahead of print. 39673465 10.1093/jxb/erae477 PMC11981899

[tpj70054-bib-0016] Dan, J. , Xia, Y. , Wang, Y. , Zhan, Y. , Tian, J. , Tang, N. et al. (2024) One‐line hybrid rice with high‐efficiency synthetic apomixis and near‐normal fertility. Plant Cell Reports, 43, 79. 38400858 10.1007/s00299-024-03154-6 PMC10894110

[tpj70054-bib-0017] D'Erfurth, I. , Cromer, L. , Jolivet, S. , Girard, C. , Horlow, C. , Sun, Y. et al. (2010) The CYCLIN‐A CYCA1;2/TAM is required for the meiosis I to meiosis II transition and cooperates with OSD1 for the prophase to first meiotic division transition. PLoS Genetics, 6, e1000989. 20585549 10.1371/journal.pgen.1000989 PMC2887465

[tpj70054-bib-0018] D'Erfurth, I. , Jolivet, S. , Froger, N. , Catrice, O. , Novatchkova, M. & Mercier, R. (2009) Turning meiosis into mitosis. PLoS Biology, 7, e1000124. 19513101 10.1371/journal.pbio.1000124 PMC2685454

[tpj70054-bib-0019] Di, S. , Zhang, P. , Zhang, J. , Liu, G. , Wang, G. , Shi, Q. et al. (2022) Tomato UVI4 homologue modulates cell expansion to participate heat‐stimulated hypocotyl elongation. Environmental and Experimental Botany, 201, 104963.

[tpj70054-bib-0020] Van Dijk, P.J. & Bakx‐Schotman, J.M.T. (2004) Formation of unreduced megaspores (Diplospory) in apomictic dandelions (Taraxacum officinale, s.l.) is controlled by a sex‐specific dominant locus. Genetics, 166, 483–492. 15020437 10.1534/genetics.166.1.483 PMC1470670

[tpj70054-bib-0021] Van Dijk, P.J. , Rigola, D. & Schauer, S.E. (2016) Plant breeding: surprisingly, less sex is better. Current Biology, 26, R122–R124. 26859270 10.1016/j.cub.2015.12.010

[tpj70054-bib-0022] Dresselhaus, T. , Sprunck, S. & Wessel, G.M. (2016) Fertilization mechanisms in flowering plants. Current Biology, 26, R125–R139. 26859271 10.1016/j.cub.2015.12.032 PMC4934421

[tpj70054-bib-0023] Figueiredo, D.D. , Batista, R.A. , Roszak, P.J. & Köhler, C. (2015) Auxin production couples endosperm development to fertilization. Nature Plants, 1, 1–6. 10.1038/nplants.2015.184 27251719

[tpj70054-bib-0024] Gilles, L.M. , Khaled, A. , Laffaire, J. , Chaignon, S. , Gendrot, G. , Laplaige, J. et al. (2017) Loss of pollen‐specific phospholipase NOT LIKE DAD triggers gynogenesis in maize. The EMBO Journal, 36, 707–717. 28228439 10.15252/embj.201796603 PMC5350562

[tpj70054-bib-0025] Goeckeritz, C.Z. , Zheng, X. , Harkess, A. & Dresselhaus, T. (2024) Widespread application of apomixis in agriculture requires further study of natural apomicts. iScience, 27, 110720. 39280618 10.1016/j.isci.2024.110720 PMC11399699

[tpj70054-bib-0026] Grossniklaus, U. , Vielle‐Calzada, J.‐P. , Hoeppner, M.A. & Gagliano, W.B. (1998) Maternal control of embryogenesis by MEDEA, a Polycomb group gene in Arabidopsis. Science, 280, 446–450. 9545225 10.1126/science.280.5362.446

[tpj70054-bib-0027] Guitton, A.‐E. , Page, D.R. , Chambrier, P. , Lionnet, C. , Faure, J.‐E. , Grossniklaus, U. et al. (2004) Identification of new members of fertilisation independent seed Polycomb group pathway involved in the control of seed development in *Arabidopsis thaliana* . Development, 131, 2971–2981. 15151989 10.1242/dev.01168

[tpj70054-bib-0028] Hands, P. , Rabiger, D.S. & Koltunow, A. (2016) Mechanisms of endosperm initiation. Plant Reproduction, 29, 215–225. 27450467 10.1007/s00497-016-0290-x PMC4978757

[tpj70054-bib-0029] Henderson, S.T. , Johnson, S.D. , Eichmann, J. & Koltunow, A.M.G. (2017) Genetic analyses of the inheritance and expressivity of autonomous endosperm formation in Hieracium with different modes of embryo sac and seed formation. Annals of Botany, 119, 1001–1010. 28130222 10.1093/aob/mcw262 PMC5604576

[tpj70054-bib-0030] Hojsgaard, D. , Klatt, S. , Baier, R. , Carman, J.G. & Hörandl, E. (2014) Taxonomy and biogeography of apomixis in angiosperms and associated biodiversity characteristics. Critical Reviews in Plant Sciences, 33, 414–427. 27019547 10.1080/07352689.2014.898488 PMC4786830

[tpj70054-bib-0031] Huang, Y. , Liang, Y. , Xie, Y. , Rao, Y. , Xiong, J. , Liu, C. et al. (2024) Efficient haploid induction via egg cell expression of dandelion PARTHENOGENESIS in foxtail millet (Setaria italica). Plant Biotechnology Journal, 22, 1797–1799. 38318962 10.1111/pbi.14302 PMC11182582

[tpj70054-bib-0032] Huang, Y. , Meng, X. , Rao, Y. , Xie, Y. , Sun, T. , Chen, W. et al. (2024) OsWUS‐driven synthetic apomixis in hybrid rice. Plant Communications, 6, 101136. 39305015 10.1016/j.xplc.2024.101136 PMC11783873

[tpj70054-bib-0033] Huo, H. , Conner, J.A. & Ozias‐Akins, P. (2009) Genetic mapping of the apospory‐specific genomic region in Pennisetum squamulatum using retrotransposon‐based molecular markers. Theoretical and Applied Genetics, 119, 199–212. 19370319 10.1007/s00122-009-1029-y

[tpj70054-bib-0034] Iwata, E. , Ikeda, S. , Matsunaga, S. , Kurata, M. , Yoshioka, Y. , Criqui, M.‐C. et al. (2011) GIGAS CELL1, a novel negative regulator of the anaphase‐promoting complex/Cyclosome, is required for proper mitotic progression and CELL fate determination in *Arabidopsis* . The Plant Cell, 23, 4382–4393. 22167058 10.1105/tpc.111.092049 PMC3269872

[tpj70054-bib-0035] Jacquier, N.M.A. , Gilles, L.M. , Pyott, D.E. , Martinant, J.‐P. , Rogowsky, P.M. & Widiez, T. (2020) Puzzling out plant reproduction by haploid induction for innovations in plant breeding. Nature Plants, 6, 610–619. 32514145 10.1038/s41477-020-0664-9

[tpj70054-bib-0036] Jefferson, R. (1994) Apomixis: a social revolution for agriculture? Biotechnology and Development Monitor, 14–16.

[tpj70054-bib-0037] Jha, P. & Kumar, V. (2018) BABY BOOM (BBM): a candidate transcription factor gene in plant biotechnology. Biotechnology Letters, 40, 1467–1475. 30298388 10.1007/s10529-018-2613-5

[tpj70054-bib-0038] Jia, H.‐H. , Xu, Y.‐T. , Yin, Z.‐J. , Qing, M. , Xie, K.‐D. , Guo, W.‐W. et al. (2023) Genome‐wide identification of the C2H2‐zinc finger gene family and functional validation of CsZFP7 in citrus nucellar embryogenesis. Plant Reproduction, 36, 287–300. 37247027 10.1007/s00497-023-00470-x

[tpj70054-bib-0039] Jia, H.‐H. , Xu, Y.‐T. , Yin, Z.‐P. , Wu, X.‐M. , Qing, M. , Fan, Y.‐J. et al. (2021) Transcriptomes and DNA methylomes in apomictic cells delineate nucellar embryogenesis initiation in citrus. DNA Research, 28, dsab014. 34424285 10.1093/dnares/dsab014 PMC8476932

[tpj70054-bib-0040] Johnston, A.J. , Matveeva, E. , Kirioukhova, O. , Grossniklaus, U. & Gruissem, W. (2008) A dynamic reciprocal RBR‐PRC2 regulatory circuit controls Arabidopsis gametophyte development. Current Biology, 18, 1680–1686. 18976913 10.1016/j.cub.2008.09.026

[tpj70054-bib-0041] Kelliher, T. , Starr, D. , Richbourg, L. , Chintamanani, S. , Delzer, B. , Nuccio, M.L. et al. (2017) MATRILINEAL, a sperm‐specific phospholipase, triggers maize haploid induction. Nature, 542, 105–109. 28114299 10.1038/nature20827

[tpj70054-bib-0042] Khanday, I. , Skinner, D. , Yang, B. , Mercier, R. & Sundaresan, V. (2019) A male‐expressed rice embryogenic trigger redirected for asexual propagation through seeds. Nature, 565, 91–95. 30542157 10.1038/s41586-018-0785-8

[tpj70054-bib-0043] Khanday, I. & Sundaresan, V. (2021) Plant zygote development: recent insights and applications to clonal seeds. Current Opinion in Plant Biology, 59, 101993. 33422964 10.1016/j.pbi.2020.101993

[tpj70054-bib-0044] Kiyosue, T. , Ohad, N. , Yadegari, R. , Hannon, M. , Dinneny, J. , Wells, D. et al. (1999) Control of fertilization‐independent endosperm development by the MEDEA polycomb gene in Arabidopsis. Proceedings of the National Academy of Sciences, 96, 4186–4191. 10.1073/pnas.96.7.4186 PMC22442 10097185

[tpj70054-bib-0045] Knudsen, K.E. , Booth, D. , Naderi, S. , Sever‐Chroneos, Z. , Fribourg, A.F. , Hunton, I.C. et al. (2000) RB‐Dependent S‐phase Response to DNA damage. Molecular and Cellular Biology, 20, 7751–7763. 11003670 10.1128/mcb.20.20.7751-7763.2000 PMC86358

[tpj70054-bib-0046] Köhler, C. , Hennig, L. , Bouveret, R. , Gheyselinck, J. , Grossniklaus, U. & Gruissem, W. (2003) Arabidopsis MSI1 is a component of the MEA/FIE Polycomb group complex and required for seed development. The EMBO Journal, 22, 4804–4814. 12970192 10.1093/emboj/cdg444 PMC212713

[tpj70054-bib-0047] Koltunow, A.M. & Grossniklaus, U. (2003) Apomixis: a developmental perspective. Annual Review of Plant Biology, 54, 547–574. 10.1146/annurev.arplant.54.110901.160842 14503003

[tpj70054-bib-0048] Lakshmanan, K.K. & Ambegaokar, K.B. (1984) Polyembryony. In: Johri, B.M. (Ed.) Embryology of Angiosperms. Berlin, Heidelberg: Springer, pp. 445–474. Available from: 10.1007/978-3-642-69302-1_9

[tpj70054-bib-0049] Lee, J.‐O. , Russo, A.A. & Pavletich, N.P. (1998) Structure of the retinoblastoma tumour‐suppressor pocket domain bound to a peptide from HPV E7. Nature, 391, 859–865. 9495340 10.1038/36038

[tpj70054-bib-0050] Li, S. , Zhou, B. , Peng, X. , Kuang, Q. , Huang, X. , Yao, J. et al. (2014) OsFIE2 plays an essential role in the regulation of rice vegetative and reproductive development. New Phytologist, 201, 66–79. 24020752 10.1111/nph.12472

[tpj70054-bib-0051] Liu, C. , He, Z. , Zhang, Y. , Hu, F. , Li, M. , Liu, Q. et al. (2023) Synthetic apomixis enables stable transgenerational transmission of heterotic phenotypes in hybrid rice. Plant Communications, 4, 100470. 36325606 10.1016/j.xplc.2022.100470 PMC10030361

[tpj70054-bib-0052] Liu, C. , Li, X. , Meng, D. , Zhong, Y. , Chen, C. , Dong, X. et al. (2017) A 4‐bp insertion at ZmPLA1 encoding a putative phospholipase a generates haploid induction in maize. Molecular Plant, 10, 520–522. 28179149 10.1016/j.molp.2017.01.011

[tpj70054-bib-0053] Liu, C. , Wang, J. , Lu, H. , Huang, Y. , Yan, H. , Liang, H. et al. (2024) Engineering synthetic apomixis in different hybrid rice varieties using the *fix* strategy. New Crops, 1, 100003.

[tpj70054-bib-0054] Liu, P. , Panda, K. , Edwards, S.A. , Swanson, R. , Yi, H. , Pandesha, P. et al. (2024) Transposase‐assisted target‐site integration for efficient plant genome engineering. Nature, 631, 593–600. 38926583 10.1038/s41586-024-07613-8 PMC11254759

[tpj70054-bib-0055] Liu, Q. , Han, D. , Cheng, D. , Chen, J. , Tian, S. , Wang, J. et al. (2024) AtRKD5 inhibits the parthenogenic potential mediated by AtBBM. Journal of Integrative Plant Biology, 66, 1517–1531. 38818961 10.1111/jipb.13678

[tpj70054-bib-0056] Luo, M. , Taylor, J.M. , Spriggs, A. , Zhang, H. , Wu, X. , Russell, S. et al. (2011) A genome‐wide survey of imprinted genes in Rice seeds reveals imprinting primarily occurs in the endosperm T. Kakutani, ed. PLoS Genetics, 7, e1002125. 21731498 10.1371/journal.pgen.1002125 PMC3121744

[tpj70054-bib-0057] Mahlandt, A. , Singh, D.K. & Mercier, R. (2023) Engineering apomixis in crops. Theoretical and Applied Genetics, 136, 131. 37199785 10.1007/s00122-023-04357-3 PMC10195744

[tpj70054-bib-0058] Marimuthu, M.P.A. , Jolivet, S. , Ravi, M. , Pereira, L. , Davda, J.N. , Cromer, L. et al. (2011) Synthetic clonal reproduction through seeds. Science, 331, 876. 21330535 10.1126/science.1199682

[tpj70054-bib-0059] Matos, J.L. , Lau, O.S. , Hachez, C. , Cruz‐Ramírez, A. , Scheres, B. & Bergmann, D.C. (2014) Irreversible fate commitment in the Arabidopsis stomatal lineage requires a FAMA and RETINOBLASTOMA‐RELATED module. eLife, 3, e03271. 25303364 10.7554/eLife.03271 PMC4225492

[tpj70054-bib-0060] Matzke, M.A. & Mosher, R.A. (2014) RNA‐directed DNA methylation: an epigenetic pathway of increasing complexity. Nature Reviews Genetics, 15, 394–408. 10.1038/nrg3683 24805120

[tpj70054-bib-0061] McClintock, B. (1956) Controlling elements and the gene. Cold Spring Harbor Symposia on Quantitative Biology, 21, 197–216. 13433592 10.1101/sqb.1956.021.01.017

[tpj70054-bib-0062] Mercier, R. , Mézard, C. , Jenczewski, E. , Macaisne, N. & Grelon, M. (2015) The molecular biology of meiosis in plants. Annual Review of Plant Biology, 66, 297–327. 10.1146/annurev-arplant-050213-035923 25494464

[tpj70054-bib-0063] Mieulet, D. , Jolivet, S. , Rivard, M. , Cromer, L. , Vernet, A. , Mayonove, P. et al. (2016) Turning rice meiosis into mitosis. Cell Research, 26, 1242–1254. 27767093 10.1038/cr.2016.117 PMC5099866

[tpj70054-bib-0064] Nakano, M. , Shimada, T. , Endo, T. , Fujii, H. , Nesumi, H. , Kita, M. et al. (2012) Characterization of genomic sequence showing strong association with polyembryony among diverse citrus species and cultivars, and its synteny with Vitis and Populus. Plant Science, 183, 131–142. 22195586 10.1016/j.plantsci.2011.08.002

[tpj70054-bib-0065] Nallamilli, B.R.R. , Zhang, J. , Mujahid, H. , Malone, B.M. , Bridges, S.M. & Peng, Z. (2013) Polycomb group gene OsFIE2 regulates Rice (Oryza sativa) seed development and grain filling via a mechanism distinct from Arabidopsis. PLoS Genetics, 9, e1003322. 23505380 10.1371/journal.pgen.1003322 PMC3591265

[tpj70054-bib-0066] Nogler, G.A. (1984) Gametophytic Apomixis. In: Johri, B.M. (Ed.) Embryology of Angiosperms. Berlin, Heidelberg: Springer, pp. 475–518. Available from: 10.1007/978-3-642-69302-1_10

[tpj70054-bib-0067] Noyes, R.D. (2007) Apomixis in the Asteraceae: diamonds in the rough. Functional Plant Science and Biotechnology, 1, 207–222.

[tpj70054-bib-0068] Nozawa, K. , Chen, J. , Jiang, J. , Leichter, S.M. , Yamada, M. , Suzuki, T. et al. (2021) DNA methyltransferase CHROMOMETHYLASE3 prevents ONSEN transposon silencing under heat stress N. M. Springer, ed. PLoS Genetics, 17, e1009710. 34411103 10.1371/journal.pgen.1009710 PMC8376061

[tpj70054-bib-0069] Ohad, N. , Margossian, L. , Hsu, Y.C. , Williams, C. , Repetti, P. & Fischer, R.L. (1996) A mutation that allows endosperm development without fertilization. Proceedings of the National Academy of Sciences, 93, 5319–5324. 10.1073/pnas.93.11.5319 PMC39243 11607683

[tpj70054-bib-0070] Ohad, N. , Yadegari, R. , Margossian, L. , Hannon, M. , Michaeli, D. , Harada, J.J. et al. (1999) Mutations in FIE, a WD Polycomb group gene, allow endosperm development without fertilization. The Plant Cell, 11, 407–415. 10072400 10.1105/tpc.11.3.407 PMC144179

[tpj70054-bib-0071] Ozias‐Akins, P. & Van Dijk, P.J. (2007) Mendelian genetics of apomixis in plants. Annual Review of Genetics, 41, 509–537. 10.1146/annurev.genet.40.110405.090511 18076331

[tpj70054-bib-0072] Pang, W. , He, W. , Liang, J. , Wang, Q. , Hou, S. , Luo, X. et al. (2024) Disruption of ClOSD1 leads to both somatic and gametic ploidy doubling in watermelon. Horticulture Research, 12(1), uhae288. Available from: 10.1093/hr/uhae288 39882171 PMC11775614

[tpj70054-bib-0073] Papikian, A. , Liu, W. , Gallego‐Bartolomé, J. & Jacobsen, S.E. (2019) Site‐specific manipulation of Arabidopsis loci using CRISPR‐Cas9 SunTag systems. Nature Communications, 10, 729. 10.1038/s41467-019-08736-7 PMC6374409 30760722

[tpj70054-bib-0074] Qi, X. , Gao, H. , Lv, R. , Mao, W. , Zhu, J. , Liu, C. et al. (2023) CRISPR/dCas‐mediated gene activation toolkit development and its application for parthenogenesis induction in maize. Plant Communications, 4, 100449. 36089769 10.1016/j.xplc.2022.100449 PMC10030315

[tpj70054-bib-0075] Qian, H. , Guo, J. & Shi, H. (2024) Genetic manipulation of the genes for clonal seeds results in sterility in cotton. BMC Plant Biology, 24, 946. 39390400 10.1186/s12870-024-05674-5 PMC11468858

[tpj70054-bib-0076] Qu, Y. , Fernie, A.R. , Liu, J. & Yan, J. (2024) Doubled haploid technology and synthetic apomixis: recent advances and applications in future crop breeding. Molecular Plant, 17, 1005–1018. 38877700 10.1016/j.molp.2024.06.005

[tpj70054-bib-0077] Quiroz, L.F. , Gondalia, N. , Brychkova, G. , McKeown, P.C. & Spillane, C. (2024) Haploid rhapsody: the molecular and cellular orchestra of *in vivo* haploid induction in plants. New Phytologist, 241, 1936–1949. 38180262 10.1111/nph.19523

[tpj70054-bib-0078] Ravi, M. & Chan, S.W.L. (2010) Haploid plants produced by centromere‐mediated genome elimination. Nature, 464, 615–618. 20336146 10.1038/nature08842

[tpj70054-bib-0079] Ren, H. , Shankle, K. , Cho, M.‐J. , Tjahjadi, M. , Khanday, I. & Sundaresan, V. (2024) Synergistic induction of fertilization‐independent embryogenesis in rice egg cells by paternal‐genome‐expressed transcription factors. Nature Plants, 10, 1892–1899. 39533074 10.1038/s41477-024-01848-z

[tpj70054-bib-0080] Rojek, J. & Ohad, N. (2023) The phenomenon of autonomous endosperm in sexual and apomictic plants. Journal of Experimental Botany, 74, 4324–4348. 37155961 10.1093/jxb/erad168 PMC10433939

[tpj70054-bib-0081] Simonini, S. , Bencivenga, S. & Grossniklaus, U. (2024) A paternal signal induces endosperm proliferation upon fertilization in Arabidopsis. Science, 383, 646–653. 38330116 10.1126/science.adj4996

[tpj70054-bib-0082] Skinner, D.J. , Mallari, M.D. , Zafar, K. , Cho, M.‐J. & Sundaresan, V. (2023) Efficient parthenogenesis via egg cell expression of maize BABY BOOM 1: a step toward synthetic apomixis. Plant Physiology, 193, 2278–2281. 37610248 10.1093/plphys/kiad461

[tpj70054-bib-0083] Song, M. , Wang, W. , Ji, C. , Li, S. , Liu, W. , Hu, X. et al. (2024) Simultaneous production of high‐frequency synthetic apomixis with high fertility and improved agronomic traits in hybrid rice. Molecular Plant, 17, 4–7. 37990497 10.1016/j.molp.2023.11.007

[tpj70054-bib-0084] Song, M. , Li, F. , Chen, Z. , Hou, H. , Wang, Y. , Liu, H. et al. (2024) Engineering high‐frequency apomixis with normal seed production in hybrid rice. iScience, 27, 111479. 39720515 10.1016/j.isci.2024.111479 PMC11667186

[tpj70054-bib-0085] Song, X. , Wang, N. , Zhou, Y. , Tian, X. , Xie, Z. , Chai, L. et al. (2024) Adventitious embryonic causal gene FhRWP regulates multiple developmental phenotypes in citrus reproduction. The Plant Journal, 119, 1494–1507. 38879817 10.1111/tpj.16870

[tpj70054-bib-0086] Sornay, E. , Forzani, C. , Forero‐Vargas, M. , Dewitte, W. & Murray, J.A.H. (2015) Activation of *CYCD 7;1* in the central cell and early endosperm overcomes cell‐cycle arrest in the Arabidopsis female gametophyte, and promotes early endosperm and embryo development. The Plant Journal, 84, 41–55. 26261067 10.1111/tpj.12957 PMC5102630

[tpj70054-bib-0087] Spillane, C. , Curtis, M.D. & Grossniklaus, U. (2004) Apomixis technology development—virgin births in farmers' fields? Nature Biotechnology, 22, 687–691. 10.1038/nbt976 15175691

[tpj70054-bib-0088] Sprunck, S. , Rademacher, S. , Vogler, F. , Gheyselinck, J. , Grossniklaus, U. & Dresselhaus, T. (2012) Egg cell–secreted EC1 triggers sperm cell activation during double fertilization. Science, 338, 1093–1097. 23180860 10.1126/science.1223944

[tpj70054-bib-0089] Thieme, M. , Lanciano, S. , Balzergue, S. , Daccord, N. , Mirouze, M. & Bucher, E. (2017) Inhibition of RNA polymerase II allows controlled mobilisation of retrotransposons for plant breeding. Genome Biology, 18, 134. 28687080 10.1186/s13059-017-1265-4 PMC5501947

[tpj70054-bib-0090] Underwood, C.J. & Mercier, R. (2022) Engineering apomixis: clonal seeds approaching the fields. Annual Review of Plant Biology, 73, 201–225. 10.1146/annurev-arplant-102720-013958 35138881

[tpj70054-bib-0091] Underwood, C.J. , Vijverberg, K. , Rigola, D. , Okamoto, S. , Oplaat, C. , den Camp, R.H.M.O. et al. (2022) A PARTHENOGENESIS allele from apomictic dandelion can induce egg cell division without fertilization in lettuce. Nature Genetics, 54, 84–93. 34992267 10.1038/s41588-021-00984-y

[tpj70054-bib-0092] Van Dijk, P.J. , Op den Camp, R. & Schauer, S.E. (2020) Genetic dissection of apomixis in dandelions identifies a dominant parthenogenesis locus and highlights the complexity of autonomous endosperm formation. Genes, 11, 961. 32825294 10.3390/genes11090961 PMC7565526

[tpj70054-bib-0093] Vernet, A. , Meynard, D. , Lian, Q. , Mieulet, D. , Gibert, O. , Bissah, M. et al. (2022) High‐frequency synthetic apomixis in hybrid rice. Nature Communications, 13(1), 7963. Available from: 10.1038/s41467-022-35679-3 PMC9794695 36575169

[tpj70054-bib-0094] Vijverberg, K. , Ozias‐Akins, P. & Schranz, M.E. (2019) Identifying and engineering genes for parthenogenesis in plants. Frontiers in Plant Science, 10, 128. Available from: 10.3389/fpls.2019.00128 30838007 PMC6389702

[tpj70054-bib-0095] Wang, C. , Liu, Q. , Shen, Y. , Hua, Y. , Wang, J. , Lin, J. et al. (2019) Clonal seeds from hybrid rice by simultaneous genome engineering of meiosis and fertilization genes. Nature Biotechnology, 37, 283–286. 10.1038/s41587-018-0003-0 30610223

[tpj70054-bib-0096] Wang, K. , Chen, H. , Ortega‐Perez, M. , Miao, Y. , Ma, Y. , Henschen, A. et al. (2021) Independent parental contributions initiate zygote polarization in *Arabidopsis thaliana* . Current Biology, 31, 4810–4816.e5. 34496220 10.1016/j.cub.2021.08.033

[tpj70054-bib-0097] Wang, N. , Song, X. , Ye, J. , Zhang, S. , Cao, Z. , Zhu, C. et al. (2022) Structural variation and parallel evolution of apomixis in citrus during domestication and diversification. National Science Review, 9, nwac114. 10.1093/nsr/nwac114. 36415319 PMC9671666

[tpj70054-bib-0098] Wang, X. , Xu, Y. , Zhang, S. , Cao, L. , Huang, Y. , Cheng, J. et al. (2017) Genomic analyses of primitive, wild and cultivated citrus provide insights into asexual reproduction. Nature Genetics, 49, 765–772. 28394353 10.1038/ng.3839

[tpj70054-bib-0099] Wang, Y. , Fuentes, R.R. , Van Rengs, W.M.J. , Effgen, S. , Zaidan, M.W.A.M. , Franzen, R. et al. (2024) Harnessing clonal gametes in hybrid crops to engineer polyploid genomes. Nature Genetics, 56, 1075–1079. 38741016 10.1038/s41588-024-01750-6 PMC11176054

[tpj70054-bib-0100] Wang, Y. & Underwood, C.J. (2023) Apomixis. Current Biology, 33, R293–R295. 37098328 10.1016/j.cub.2023.01.051

[tpj70054-bib-0101] Wei, X. , Liu, C. , Chen, X. , Lu, H. , Wang, J. , Yang, S. et al. (2023) Synthetic apomixis with normal hybrid rice seed production. Molecular Plant, 16, 489–492. 36609144 10.1016/j.molp.2023.01.005

[tpj70054-bib-0102] Weinberg, R.A. (1995) The retinoblastoma protein and cell cycle control. Cell, 81, 323–330. 7736585 10.1016/0092-8674(95)90385-2

[tpj70054-bib-0103] Wu, X. , Xie, L. , Sun, X. , Wang, N. , Finnegan, E.J. , Helliwell, C. et al. (2023) Mutation in Polycomb repressive complex 2 gene OsFIE2 promotes asexual embryo formation in rice. Nature Plants, 9, 1848–1861. 37814022 10.1038/s41477-023-01536-4 PMC10654051

[tpj70054-bib-0104] Xiong, J. , Hu, F. , Ren, J. , Huang, Y. , Liu, C. & Wang, K. (2023) Synthetic apomixis: the beginning of a new era. Current Opinion in Biotechnology, 79, 102877. 36628906 10.1016/j.copbio.2022.102877

[tpj70054-bib-0105] Yadav, C.B. , Rozen, A. , Eshed, R. , Ish‐Shalom, M. , Faigenboim, A. , Dillon, N. et al. (2023) Promoter insertion leads to polyembryony in mango — a case of convergent evolution with citrus. Horticulture Research, 10, uhad227. 38077495 10.1093/hr/uhad227 PMC10709545

[tpj70054-bib-0106] Ye, H. , Louden, M. & Reinders, J.A.T. (2024) A novel in vivo genome editing doubled haploid system for Zea mays L. Nature Plants, 10, 1493–1501. 39333351 10.1038/s41477-024-01795-9

[tpj70054-bib-0107] Yin, P.P. , Tang, L.P. , Zhang, X.S. & Su, Y.H. (2022) Options for engineering apomixis in plants. Frontiers in Plant Science, 13, 864987. Available from: 10.3389/fpls.2022.864987 35371148 PMC8967160

[tpj70054-bib-0108] Zhang, C. , Yang, Z. , Tang, D. , Zhu, Y. , Wang, P. , Li, D. et al. (2021) Genome design of hybrid potato. Cell, 184, 3873–3883.e12. 34171306 10.1016/j.cell.2021.06.006

[tpj70054-bib-0109] Zhang, Z. , Conner, J. , Guo, Y. & Ozias‐Akins, P. (2020) Haploidy in tobacco induced by PsASGR‐BBML transgenes via parthenogenesis. Genes, 11, 1072. 32932590 10.3390/genes11091072 PMC7564442

[tpj70054-bib-0110] Zhong, Y. , Liu, C. , Qi, X. , Jiao, Y. , Wang, D. , Wang, Y. et al. (2019) Mutation of ZmDMP enhances haploid induction in maize. Nature Plants, 5, 575–580. 31182848 10.1038/s41477-019-0443-7

[tpj70054-bib-0111] Zuo, J. , Niu, Q. , Frugis, G. & Chua, N. (2002) The *WUSCHEL* gene promotes vegetative‐to‐embryonic transition in *Arabidopsis* . The Plant Journal, 30, 349–359. 12000682 10.1046/j.1365-313x.2002.01289.x

